# Study of molecular patterns associated with ferroptosis in Parkinson’s disease and its immune signature

**DOI:** 10.1371/journal.pone.0295699

**Published:** 2023-12-21

**Authors:** Lixia Chen, Guanghao Xin, Yijie He, Qinghua Tian, Xiaotong Kong, Yanchi Fu, Jianjian Wang, Huixue Zhang, Lihua Wang

**Affiliations:** 1 Department of Neurology, The Second Affiliated Hospital of Harbin Medical University, City Harbin, Province Heilongjiang, China; 2 Department of Neurology, The 962 Hospital of the Chinese People’s Liberation Army Joint Logistic Support Force, City Harbin, Province Heilongjiang, China; The University of Texas at El Paso, UNITED STATES

## Abstract

Parkinson’s disease is the second most common neurodegenerative disease in the world. We downloaded data on Parkinson’s disease and Ferroptosis-related genes from the GEO and FerrDb databases. We used WCGAN and Random Forest algorithm to screen out five Parkinson’s disease ferroptosis-related hub genes. Two genes were identified for the first time as possibly playing a role in Braak staging progression. Unsupervised clustering analysis based on hub genes yielded ferroptosis isoforms, and immune infiltration analysis indicated that these isoforms are associated with immune cells and may represent different immune patterns. FRHGs scores were obtained to quantify the level of ferroptosis modifications in each individual. In addition, differences in interleukin expression were found between the two ferroptosis subtypes. The biological functions involved in the hub gene are analyzed. The ceRNA regulatory network of hub genes was mapped. The disease classification diagnosis model and risk prediction model were also constructed by applying hub genes based on logistic regression. Multiple external datasets validated the hub gene and classification diagnostic model with some accuracy. This study explored hub genes associated with ferroptosis in Parkinson’s disease and their molecular patterns and immune signatures to provide new ideas for finding new targets for intervention and predictive biomarkers.

## 1 Introduction

Parkinson’s disease (PD) is the second largest neurodegenerative disease in the world, mainly manifested by motor and non-motor symptoms, gradually losing the ability to work and live in the late stage, causing a huge burden to society and families [1]. More than 6 million people worldwide had PD in 2016, and the incidence is gradually increasing [2]. PD is expected to reach 10 million people by 2030 [3]. The exact pathogenesis and causative factors of Parkinson’s disease are unknown. The primary pathological process can be summarized as "one increase and one decrease". "One decrease" is the degenerative death of dopaminergic neurons in dense areas of the substantia nigra(SN), and "One increase" means that Lewy bodies, mainly α-synuclein(α-syn), appear in the remaining neuron cytoplasm [4, 5]. In the past, the degenerative death process of PD dopaminergic neurons has been recognized as apoptosis [6]. With the discovery of multiple modes of programmed cell death, including ferroptosis and cellular pyroptosis, the discussion on the mode of cell death and pathogenesis of PD has been enriched [7].

Ferroptosis is a new mode of programmed cell death identified by Dixon et al. in 2012 [8]. Ferroptosis’s hallmark events manifest as iron-dependent and lipid peroxidation(LPO) [8]. Multiple cellular metabolic pathways regulate this mode of death, as well as various disease-related signaling pathways [9]. Specifically, the main manifestations are an imbalance in iron homeostasis, production of reactive oxygen species (ROS) due to LPO, depletion of glutathione (GSH), inactivation of glutathione peroxidase 4(GPX4) in cells, and changes in the expression levels of a unique set of regulatory genes [8, 10].

The current study also identified several metabolic pathways associated with the ferroptosis inhibition system. The System XC-/GSH/GPX4 axis was the earliest identified pathway regulating ferroptosis and is considered the major system counteracting ferroptosis in mammals [11]. The NAD(P)H/FSP1/CoQ10 axis(Ferroptosis suppressor protein 1, FSP1) is a second ferroptosis regulatory pathway with a different site of action from the GPX4 axis that can continue to function as an inhibitor of ferroptosis in GPX4-deficient cells [12, 13]. GTP hydrolase-1 (GCH1) is the rate-limiting enzyme for tetrahydrobiopterin (BH4) synthesis, while GCH1 is another ferroptosis repressor gene that is not dependent on GPX4 [14, 15]. The DHODH/CoQ-H2 axis is the most recently discovered ferroptosis inhibitory pathway. Dihydroorotate Dehydrogenase (DHODH), located on the outer surface of the inner mitochondrial membrane, inhibits mitochondrial LPO in a coenzyme Q10-dependent manner by reducing ubiquinone to form CoQ10-H2, thereby blocking ferroptosis of mitochondrial inner membrane origin [14].

It is hypothesized that ferroptosis may be one of the most prevalent and oldest forms of cell death [16]. Although initially studied in mammalian systems, ferroptosis-like cell death has also been observed in evolutionarily distant species, such as those belonging to the phyla, protozoa and fungi [17–23]. Over the past decade of research, ferroptosis has been associated with the pathogenesis of various diseases involving virtually every organ and system in the body, including various cancers, neurodegenerative diseases, cardiovascular diseases, respiratory diseases, and autoimmune diseases [24]. Among other things, ferroptosis has been found to be associated with stroke, neurodegenerative diseases (Alzheimer’s disease, Huntington’s disease, PD), multiple sclerosis (MS), amyotrophic lateral sclerosis (ALS), and other neurological disorders [6, 25–29].

Ferroptosis has been shown to be a common type of cell death in PD [30]. The pathologic features of PD are highly overlapping with key features and triggers of the ferroptosis pathway, including aberrant iron accumulation, LPO, reduced levels of GSH and System XC- levels, and reduced DJ-1 and CoQ10 [9, 31, 32]. In studies in cell models, in vitro brain slice cultures, dopaminergic neurons (DNs)were found to be sensitive to the classical ferroptosis inducer erastin. In contrast, the ferroptosis-specific inhibitors Ferrostatin-1(Fer-1) and Liproxstatin-1(Lip-1), as well as iron chelator, inhibited the death of DNs. In addition, these inhibitors prevented the cell death associated with sporadic PD induced by rotenone, paraquat, and MPP+(1-methyl-4-phenylpyridinium). Similarly, ferroptosis was shown to characterize the MPTP(1-Methyl-4-phenyl-1,2,3,6-tetrahydropyridine)-induced PD mouse model and Fer-1 and Lip-1 prevented the loss of DNs in the SN and striatum, as well as motor deficits [30]. In addition, a recent study found that arachidonic acid (AA) +Fe^3+^ treatment of human dopaminergic neurons differentiated from the LUHMES cell line decreased intracellular GSH levels, survival, and increased LPO and 4-hydroxynonenal. This trend was attenuated by deferoxamine and Lip-1 [33]. These studies further confirm support for the presence of ferroptosis in DNs in PD, and it is promising to study the role of ferroptosis in PD. Inhibition of ferroptosis may attenuate the symptoms of PD or even prevent the onset of PD. Abnormal iron accumulation is thought to characterize the pathology of the SN in PD patients [34]. In addition to this, mutations or knockouts of divalent metal transporter 1(DMT1), ferritin, Ferroportin (FPN), and β-amyloid precursor protein in PD animal models and human brain SN cause abnormal disturbances in iron uptake and output, leading to the formation of free radicals to promote ferroptosis to occur [34–39]. In response to the reality of iron deposition, iron chelating agents have become a practical therapeutic pair [40]. It restored MPTP-induced iron deposition in the mouse brain to normal levels. Still, more importantly, it reduced iron deposition in the SN of patients and slowed down the rate of symptom progression [40, 41].

In addition, PD may be associated with neuroinflammation and autoimmunity. It has been found that α-syn can act as a beacon for specific T cells, causing them to attack brain cells mistakenly and possibly contributing to the progression of PD [42]. However, the exact mechanism is unclear. Studies from cancer found that CD8+ T cells can exert their tumor suppressive effects by secreting interferon-gamma (IFNγ) to mediate the downregulation of SLC7A11(Recombinant Solute Carrier Family 7, Member 11)-triggered ferroptosis in tumor cells [43, 44]. In addition, IFNγ also upregulates Acyl-CoA Synthetase Long chain family member 4(ACSL4), which contributes to synthesizing the ferroptosis substrate PL-PUFA (Phospholipid-Polyunsaturated Fatty Acids). Detecting excess AA in the tumor microenvironment suggests that AA + IFNγ derived from CD8+ T cells may be the first identified natural ferroptosis trigger [45]. Follicular CD4+ helper T cells (Tfh) can promote B-cell responses for persistent immunity. Tfh cell numbers are regulated by GPX4-controlled cell death, and increasing GPX4 abundance through selenium supplementation may increase antibody responses after influenza vaccination [46]. This evidence suggests that neuroimmune may be physiologically or pathologically regulated through the ferroptosis pathway.

Currently, there is no way to slow or stop the progression of PD. Determining the specific mechanism of ferroptosis in Parkinson’s disease is expected to lead to the development of new therapeutic targets for PD, slowing or even reversing disease progression. In addition, although criteria for diagnosing PD precursors have been proposed in 2019, they are unsuitable for large-scale application [47]. Ferroptosis studies based on pathogenic mechanisms will identify new biomarkers and provide new ideas for large-scale PD screening and prevention. This study aimed to comprehensively analyze and explore the molecular mechanisms of FRGs in the pathogenesis of PD and the immunological features using transcriptomics data and to provide evidence from genetic and algorithmic sources for discovering new therapeutic modalities and biomarkers. We put the abbreviations and corresponding explanations of this article in Table 1.

**Table 1:** List of abbreviations.

Abbreviation	Full name	Abbreviation	Full name
WGCNA	Weighted Gene Co-expression Network Analysis	ACSL4	Acyl-CoA Synthetase Long chain family member 4
FRHGs	Ferroptosis-related hub genes	GSVA	Gene Set Variation Analysis
ceRNA	competing endogenous RNA	PUFAs	Polyunsaturated Fatty Acids
PD	Parkinson’s disease	PCA	Principal Component Analysis
SN	substantia nigra	RF	Random Forest
α-syn	α-synuclein	ROC	Receiver operating characteristic curve
ROS	Reactive Oxygen Species	PL	Phospholipid
LPO	Lipid Peroxidation	SD	standard deviation
GSH	glutathione	DNs	dopaminergic neurons
GPX4	glutathione peroxidase 4	AUC	Area Under the Curve
ME	Module Eigengene	GO	Gene Ontology
BP	Biological Process	AA	Arachidonic acid
CC	Cellular Component	Tfh	helper T cells
MF	Molecular Function	Lip-1	Liproxstatin-1
OS	Oxidative stress	Fer-1	Ferrostatin-1
FSP1	ferroptosis suppressor protein 1	FPN	Ferroportin
GCH1	GTP hydrolase-1	DMT1	Divalent metal transporter 1
BH4	tetrahydrobiopterin	CoQ10	Coenzyme Q10
DHODH	Dihydroorotate Dehydrogenase	MS	multiple sclerosis
NAD(P)H	nicotinamide adenosine dinucleotide (phosphate)	MPTP	1-Methyl-4-phenyl-1,2,3,6-tetrahydropyridine
SVM	Support Vector Machines	GESA	Gene Set Enrichment Analysis
SLC7A11	Recombinant Solute Carrier Family 7, Member 11	KEGG	Kyoto encyclopedia of genes and genomes
SNX4	Sorting Nexin 4	DA	Dopamine
SNX5	Sorting Nexin 5	MPP+	1-methyl-4-phenylpyridinium
ssGSEA	single sample Gene Set Enrichment Analysis		
D-PUFAs	deuterated PUFAs	GS	Gene Significance
FRGs	ferroptosis-related genes	MM	Module Membership
System XC-	Cystine/glutamate antiporter	ALS	Amyotrophic lateral sclerosis

## 2 Results

### 2.1 Part one: Acquisition of ferroptosis-related hub genes in Parkinson’s disease

#### Obtain the merged dataset

**Fig 1:**
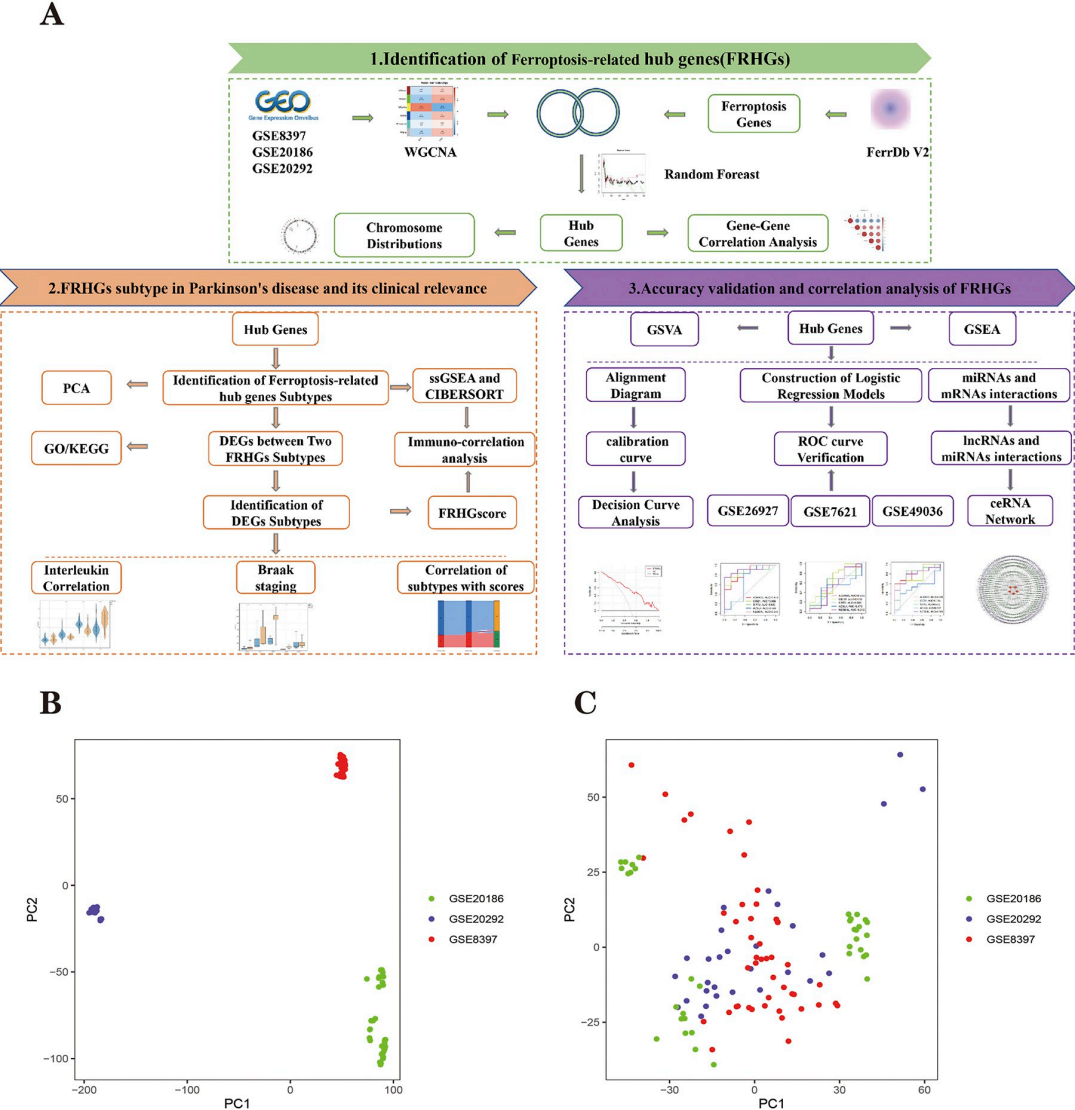
**(A)** Technology roadmap for this study. **(B)** PCA plot when the data set is merged and not batch corrected. **(C)** PCA plot after batch correction. Each dot in the PCA diagram represents a sample, where the red dot represents the distribution of GSE8397 samples, the purple dot represents the distribution of GSE20292 samples, and the green dot represents the distribution of GSE20186 samples.

An expression matrix containing 11,894 genes from 47 healthy individuals and 49 nigrostriatal tissues of Parkinson’s disease patients was obtained by merging the three datasets. A principal component analysis (PCA)plot of the merged dataset with the batch effect removed is plotted, and Fig 1B shows that the merged dataset is aggregated in different regions with the batch effect separately. Fig 1C shows that after batch correction, the three dataset samples are mixed at one time, mitigating the batch effect.

#### Obtain two modules related to PD

**Fig 2:**
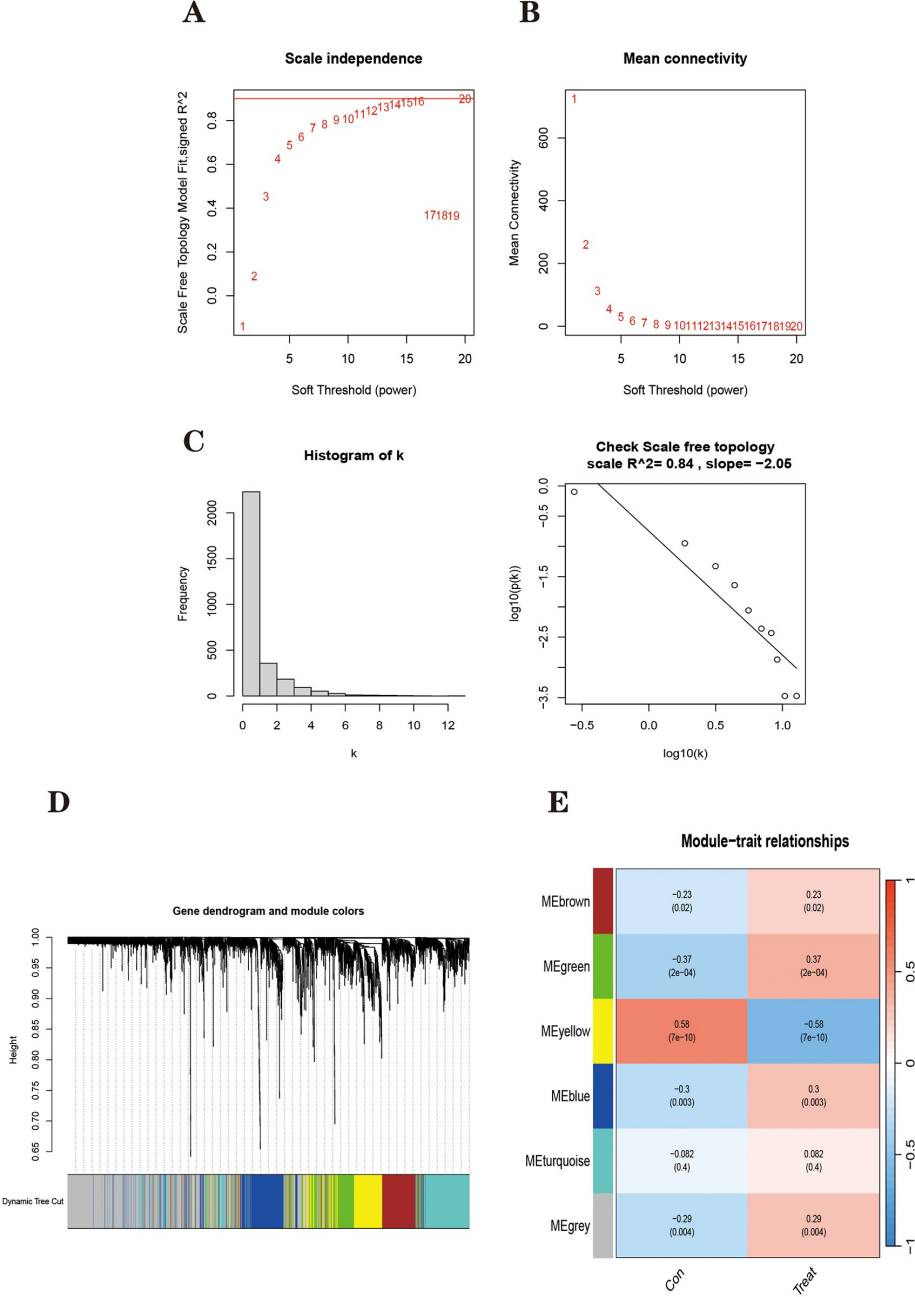
Analysis of weighted co-expression networks in Parkinson’s disease. **(A)** Scale-free index for analyzing the power of various soft thresholds. The horizontal coordinate represents the power of soft thresholds, and the best soft threshold is marked with a red line. **(B)** Average connectivity of various soft thresholds. **(C)** Check whether the set soft threshold satisfies the scale-free network. **(D)**Identification of co-expressed gene modules. A dendrogram of all differentially expressed genes was clustered based on a measure of gene similarity. **(E)** Heat map of the correlation between modules and clinical phenotypes. The corresponding cor value and *P* value are labeled therein. The yellow modules have the strongest correlation with PD.

A total of 49 PDs, 47 healthy control samples, and their 11,894 genes were included in the Weighted Gene Co-expression Network Analysis (WGCNA). Considering that most of the 11,894 genes show little difference in expression levels across samples, their contribution to the co-expression network construction is limited, and too many genes would consume computational resources [48, 49]. After referring to a large number of literature sources, to ensure the successful construction of the co-expression network while reducing the computational workload, we chose to calculate the top 25% of the standard deviation (SD) ranking for each gene (2974 genes) to proceed with constructing the co-expression network [50–52]. Ultimately, 2974 genes from 96 samples were used to construct a weighted gene co-expression network. After calculation, the optimal soft threshold was determined to be 13 (Fig 2A and 2B). To check whether the optimal soft threshold satisfies the scale-free network, the R2 value of the model was calculated to be 0.84, which indicates that the gene association is more consistent with the scale-free distribution (Fig 2C). Subsequently, this adjacency matrix is transformed into a TOM matrix (topological overlap matrix), which better reflects the connection and adjacency relationships between genes. The final 6 modules were obtained (Fig 2D).

Correlation heat maps were drawn for the six modules and clinical traits (Fig 2E). In this case, the Gray modules are used to place genes that do not belong to any module and are not considered to be of clinical analysis value.

**Fig 3:**
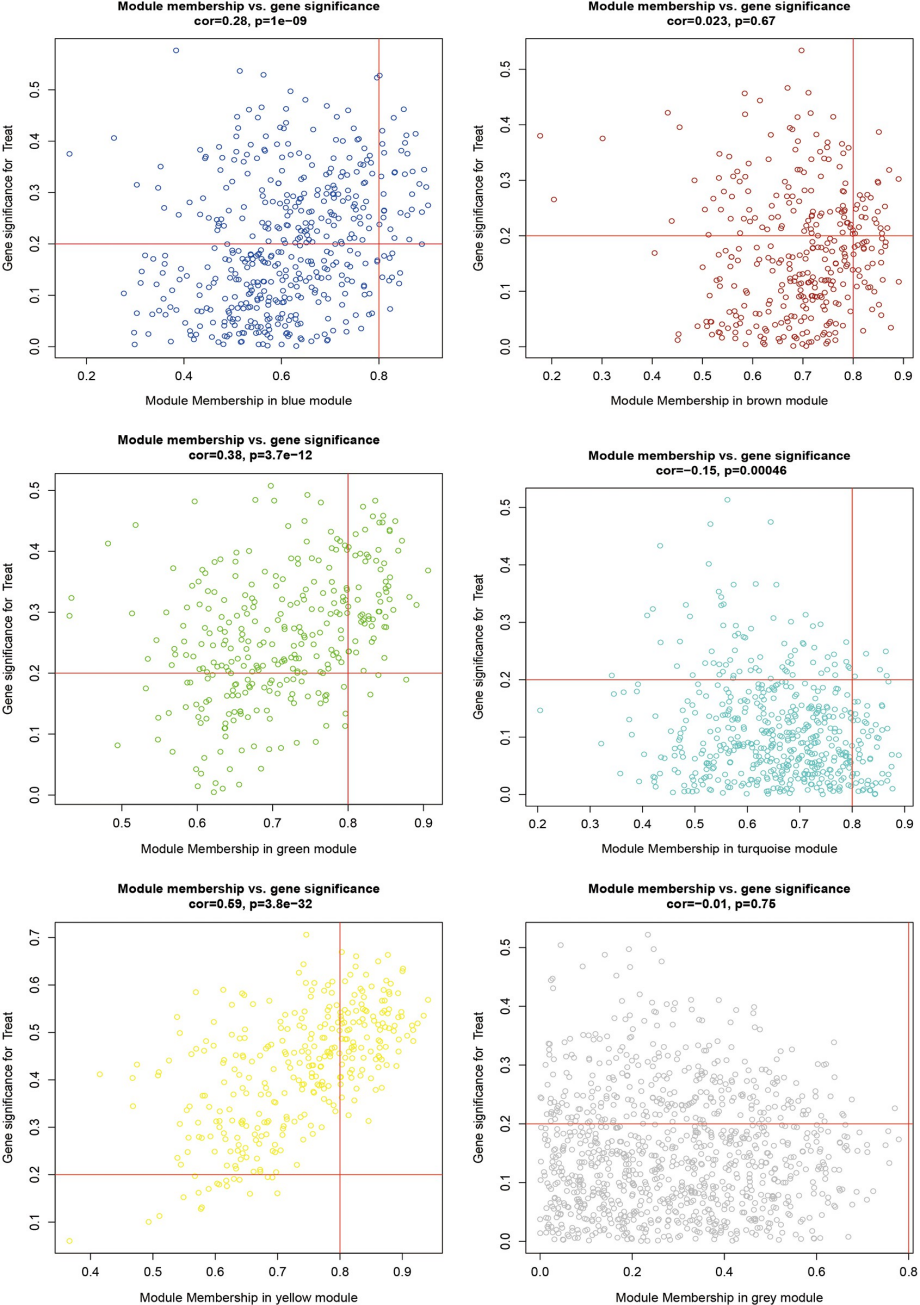
Scatter plot of correlation between Module Membership (MM) and Gene Significance (GS).

The Pearson correlation coefficient was then used to calculate the relationship between gene expression levels and clinical performance for the remaining five modules. A scatter plot of Gene Significance (GS) and Module Membership (MM) correlations was plotted (Fig 3). Based on the clinical trait correlation heat map it can be visualized that the yellow module had the highest negative clinical correlation with the PD group; the Green module (cor = 0.37, p<2e-04) had the highest positive clinical correlation with the PD group. Also, the scatter plot showed that the correlation between MM and GS was also highest for these two modules, indicating that these genes, which are highly correlated with traits, also play the role of pivotal genes in the key modules. These two modules will be analyzed further. Among them, the yellow module contains 328 genes and the Green module contains 312 genes.

#### Annotation of the biological functions of positive and negative correlation modules in Parkinson’s disease

Gene Ontology (GO) and Kyoto encyclopedia of genes and genomes (KEGG) enrichment analyses were performed to further explore the functions and signaling pathways that may be involved in the positive and negative modules associated with PD. The GO analysis illustrates the functions enriched by the module at three levels: Biological Process (BP), Cellular Component (CC), and Molecular Function (MF).

**Fig 4:**
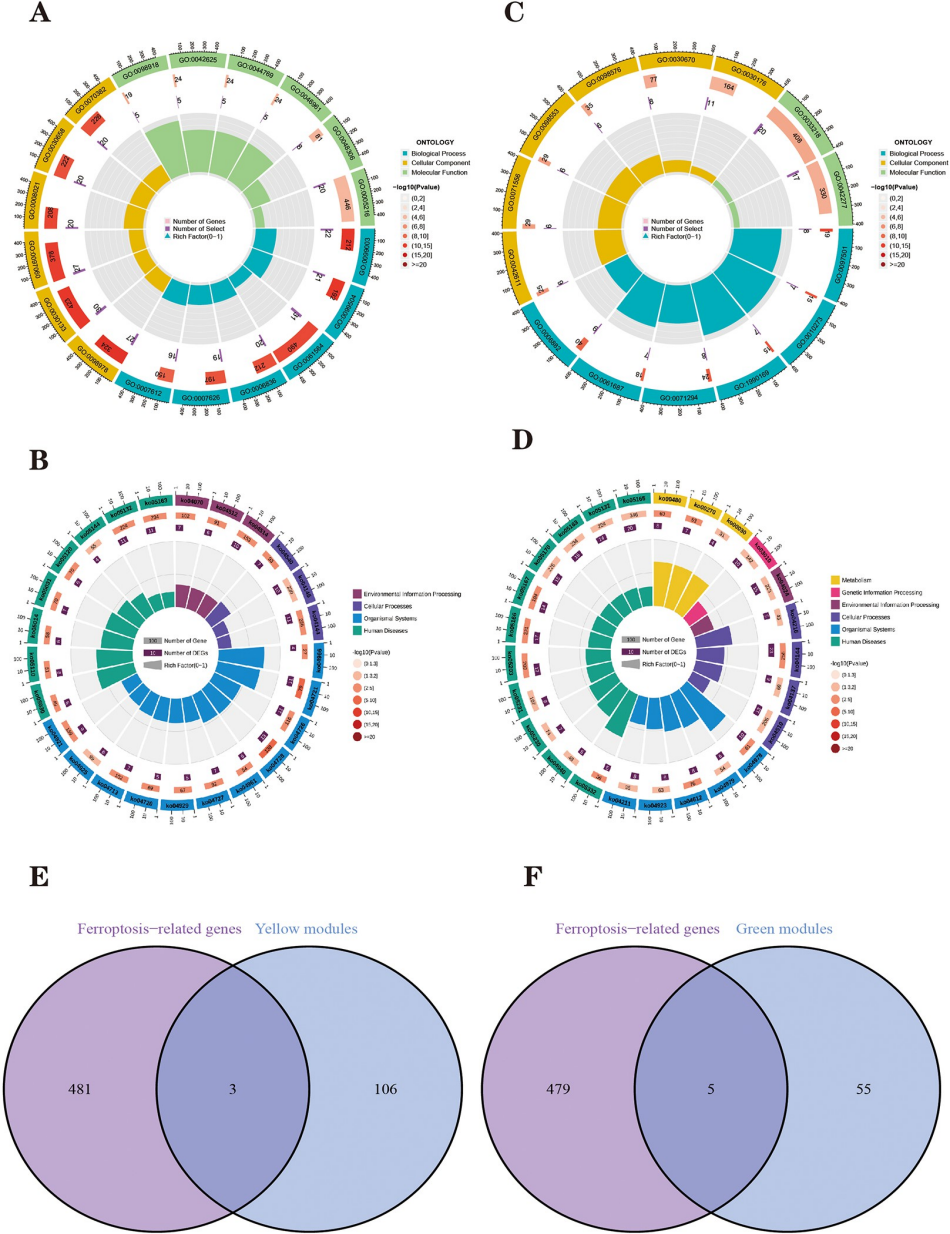
**(A, B)** The GO enrichment and KEGG pathway analysis results of yellow module are shown as circle plots. **(C, D)** The GO enrichment and KEGG pathway analysis results of yellow module are shown as circle plots. For enrichment analysis of the circle plot, the GO id (or pathway id) label of the first circle corresponds to the “id” of the result data (S1 Data (or S2 Data)), and the “class” of the result data corresponds to the color of the grouping. The length of the bar in the second circle corresponds to the “bg_num” of the resulting data, i.e., the number of background genes, and the shade of the colour corresponds to the P value (or Q value). The third circle corresponds to the “fg_num” of the resulting data, i.e., the number of foreground genes. The fourth circle (polar bar) shows the Rich factor, obtained by dividing fg_num and bg_num and corresponds to the data in the ratio column of “S1 Data (or S2 Data)”. **(E)** The yellow module is taken to intersect with ferroptosis-related genes. **(F)** The green module is taken to intersect with Ferroptosis-related genes.

The yellow module is the most negatively related module to PD, where BP-related functions are mainly related to “vesicle-mediated transport in synapse”, “synaptic vesicle cycle”, “axon development”, “learning”, and “locomotory behaviour”. CC-related functions are mainly related to “synapse”, “neuronal cell body”, “exocytic vesicle” and “transport vesicle”. MF-related functions are mainly related to synaptic structure, “ion channel activity”, “proton-transporting ATPase activity”, “rotational mechanism”, “calcium-dependent protein binding” and “ATPase-coupled ion transmembrane transporter activity” (Fig 4A). See S1 Data for details. KEGG analysis revealed that the Yellow module is involved in PD development mainly through the “Phosphatidylinositol signaling system”, “Endocytosis”, “Phagosome”, “Synaptic vesicle cycle”, “Dopaminergic synapse” and “Amphetamine addiction” pathways are involved in PD development (Fig 4B). We can find that the functions of these modules are related to the development of PD occurrence. See S2 Data for details.

The green module is the most positively correlated module with PD, where BP-related functions are mainly related to the cellular response to metal ions, “stress response to copper ion”, “cellular response to zinc ion” and “detoxification of inorganic compound”. CC-related functions are mainly associated with the “endoplasmic reticulum membrane”, “phagocytic vesicle membrane” and “MHC protein complex”. The MF-related functions are mainly related to “amide binding” and “peptide binding” (Fig 4C). See S3 Data for details. The Green module is mainly involved in “glutathione metabolism”, “Pentose phosphate pathway”, “Cysteine and methionine metabolism”, “cAMP signaling pathway”, “Ribosome”, “Endocytosis”, “Ferroptosis”, “Mineral absorption”, “Antigen processing and presentation” and “Viral carcinogenesis” pathways are involved in PD development (Fig 4D). See S4 Data for details. This module is enriched for functions related to PD developmental functions and pathways, but also seen to be enriched for ferroptosis pathways and pathways related to ferroptosis such as glutathione, cysteine and methionine metabolism, and mineral uptake. In addition, with antigen processing presentation function is also enriched by this module.

#### Acquisition of ferroptosis-related genes in Parkinson’s disease and their correlation with Braak staging

**Fig 5:**
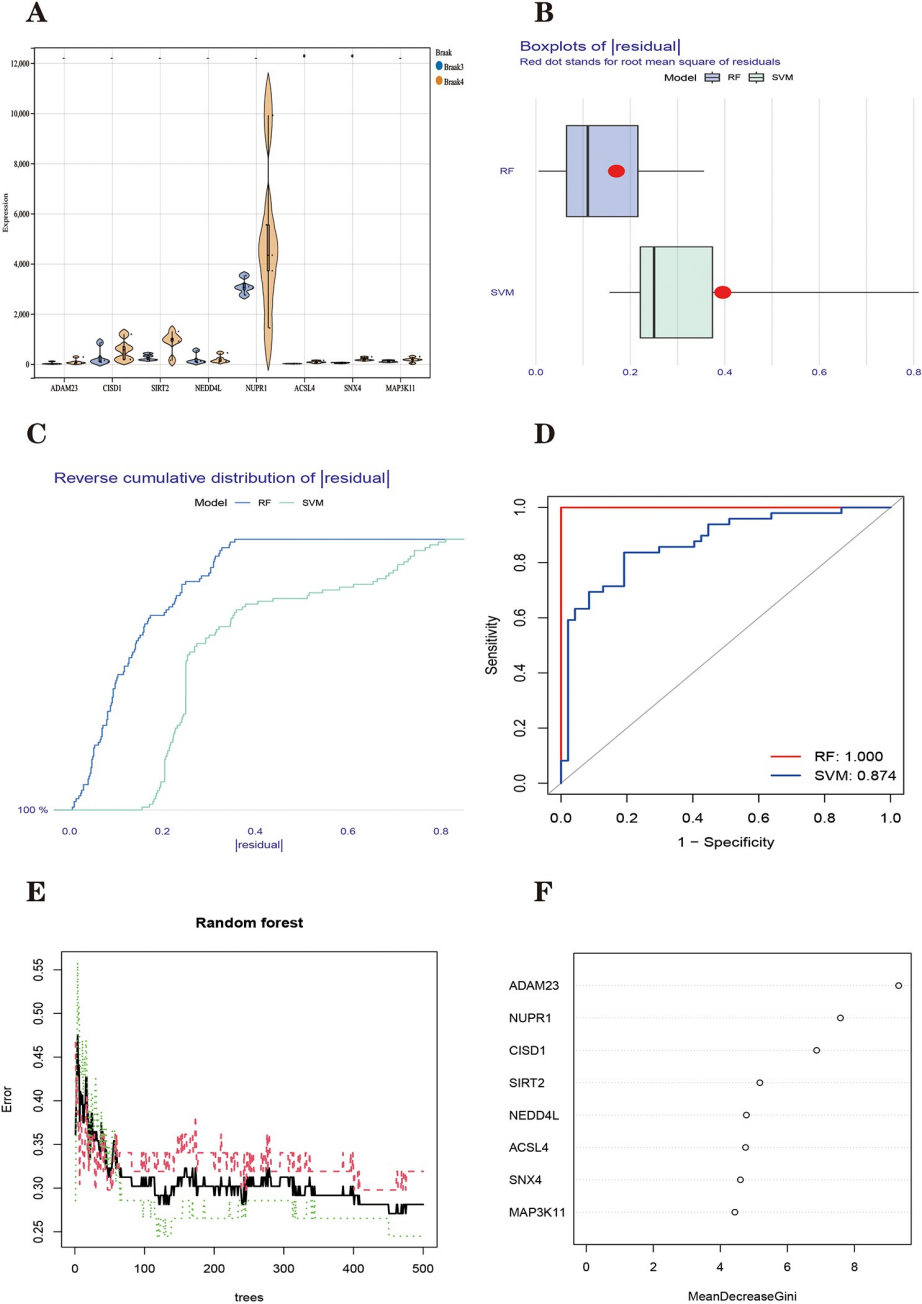
**(A)** Violin diagram depicting the expression of ferroptosis-related genes in Braak3 and Braak4 stages. The X-axis represents the gene and the Y-axis represents the amount of expression. **(B)** Boxplots of residual. The red dots represent the mean and the box plot represents the quantile. **(C)** Reverse cumulative distribution of residual. **(D)** The AUC value of the ROC curve indicated that the RF model (1.000) has higher accuracy than the SVM model (0.874). **(E)** Plot of decision tree versus error. The x-axis represents the number of decision trees; the y-axis represents the error. **(F)** Screening of hub genes by Gini coefficient method. The X-axis represents the importance index, the y-axis represents the FRGs, and all FRGs are ranked according to the “mean reduction Gini coefficient.” The higher the value, the closer the relationship between the gene and the disease. *P < 0.05; **P < 0.01; ****P < 0.001.

WGCNA can screen for hub genes within phenotype-related modules and obtain phenotype-related modules [48, 49]. The essence of Gene Significance (GS) is the correlation between the genes within the module where it is located and the phenotype (our phenotype was selected as whether or not we had PD) [48]. Reviewing a large amount of literature, we found that, generally, a GS threshold between 0.2 and 0.5 is reasonable [53–60]. At the same time, we wanted to include as many genes as possible to see the relationship with ferroptosis, so for the GS threshold, we chose 0.2. Module Membership (MM) stands for the correlation between each gene in a module and the module in which it is located, indicating whether it is consistent with the trend of the module [48]. Whereas the MM threshold used to screen for pivotal genes should be high to retain those genes that are closely related to their modules, the threshold is usually between 0.7 and 0.9. 0.8 is the most common choice of threshold [61–64]. Based on the thresholds of GS>0.2 and MM>0.8, we extracted 109 key genes from the Yellow module and 60 key genes from the Green module. A total of 728 genes were downloaded from the FerrDb database, and the number of FRGs was 484 after the removal of duplicates. The key genes of each of these two modules intersected with FRGs. The Yellow module yielded three FRGs in Parkinson’s disease (CISD1, ADAM23 and NEDD4L) (Fig 4E). Green module was obtained for five Parkinson’s disease FRGs (MAP3K11, SNX4, SIRT2, NUPR1, and ACSL4) (Fig 4F). We extracted the expression of these eight Parkinson’s disease FRGs and constructed an expression matrix of Parkinson’s disease FRGs. The correlation between Braak staging of Parkinson’s disease and FRHGs in the GSE42966 dataset was analyzed using the nonparametric Wilcoxon rank sum test, and Fig 5A shows that ACSL4 is differentially expressed with SNX4 in Braak3 and Braak4 grading.

#### Analysis of the acquisition of ferroptosis-related hub genes in Parkinson’s disease and their interactions

Random forest (RF) and Support Vector Machines (SVM) models were developed to select key Parkinson’s disease ferroptosis-related hub genes (FRHGs). Fig 5B shows that the RF model has smaller residuals than the SVM model. The curves represented by the RF model are seen in Fig 5C to reach probability 1 faster than those represented by the SVM model. The receiver operating characteristic (ROC) curves of these two models show that the area under the ROC curve of RF is 1.000, which is more accurate than that of SVM (AUC = 0.874) (Fig 5D). All three methods show that most of the samples in the RF model have relatively small residuals, indicating that the model is better.

Screening for FRHGs in Parkinson’s disease using random forest screening. Ranking of genes according to importance. The top 5 genes were extracted as FRHGs in Parkinson’s disease (CISD1, SIRT2, NUPR1, ADAM23 and NEDD4L) (Fig 5E and 5F).

**Fig 6:**
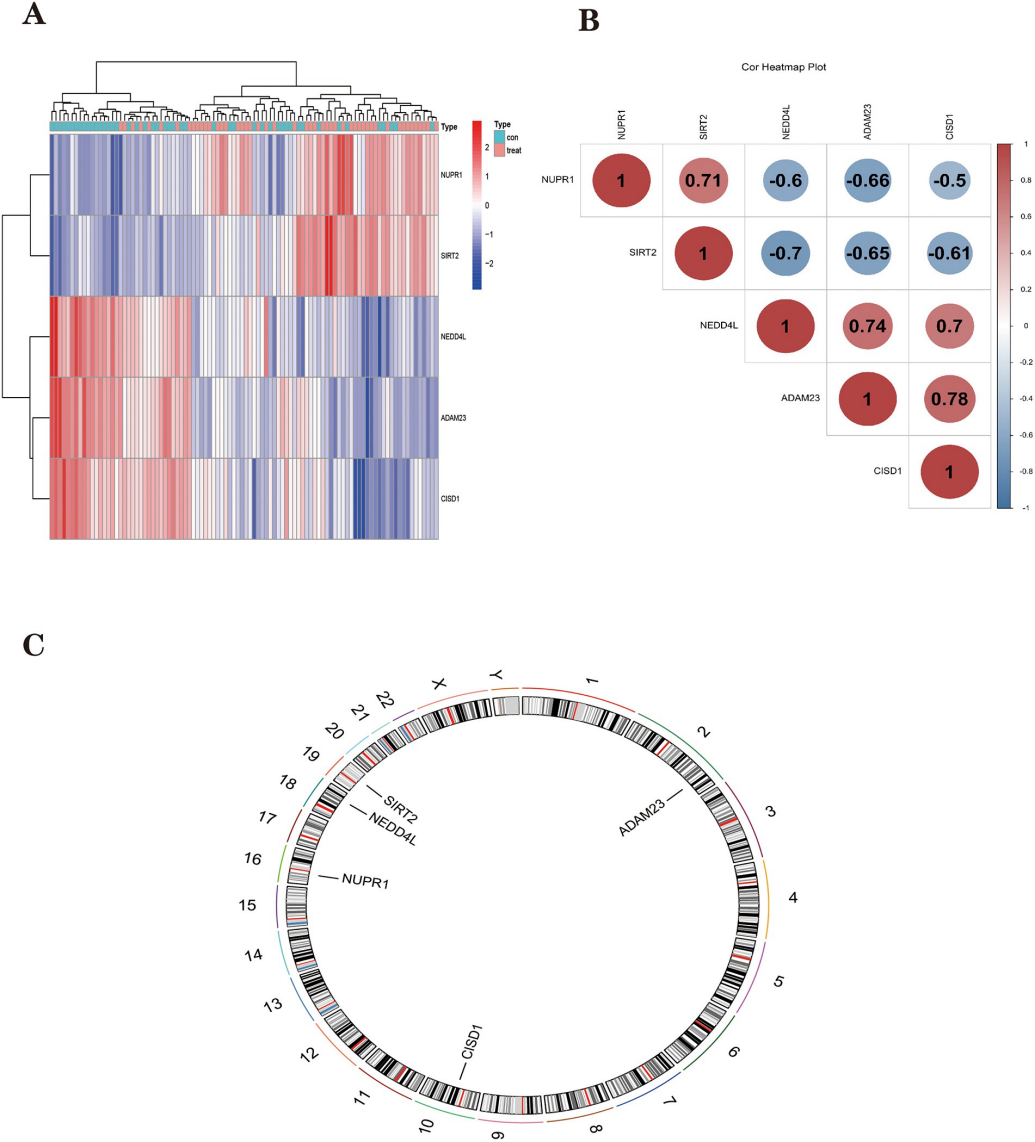
PD hub genes analysis. **(A)** The clustering heat map shows the clustering results of the five FRHGs screened by the random forest algorithm in the GSE63060 dataset. The red color represents the highly expressed genes in the samples, the blue color represents the lowly expressed genes in the samples, the blue color at the top of the heat map represents the control group samples, and the red color represents the treat group (PD)samples. **(B)** Heatmap of correlations for Hub genes. Positive correlations are marked in red and negative correlations are marked in blue. The numbers in the middle represent correlation coefficients. **(C)** Map of the location of the Hub gene on the chromosome.

The Parkinson’s disease dataset was clustered and heatmaps were drawn (Fig 6A), and these five Parkinson’s disease FRHGs were found to be effective in differentiating between PD and healthy populations. The location of the distribution of these five Parkinson’s disease FRHGs on the chromosome was analyzed (Fig 6C). Correlations between FRHGs in Parkinson’s disease were calculated using Pearson correlation coefficients and revealed a close association between FRHGs that may interact in the pathogenic process of PD. To make this correlation easier to understand, a correlation heat map (Fig 6B) was drawn using the Hiplot (https://hiplot.com.cn) online website.

### 2.2 Part two: Ferroptosis-related hub gene subtype in Parkinson’s disease and its clinical relevance

#### Ferroptosis-related hub genes in Parkinson’s disease mediate two distinct subtypes of immune infiltration

**Fig 7:**
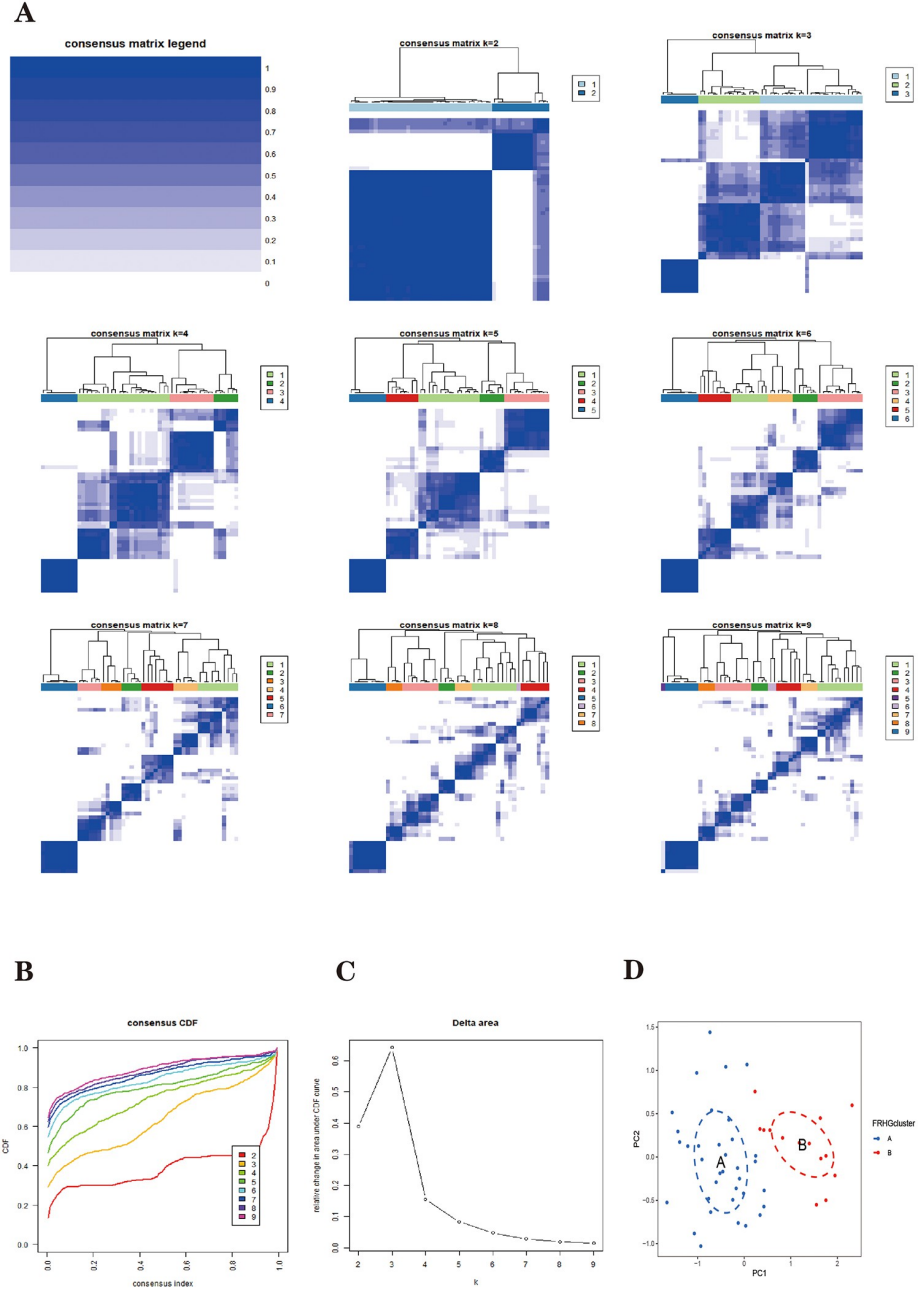
The results of two subtypes of patients with PD. **(A)** The first plot indicates the color gradient from 0 to 1 (white: 0, blue: 1). The 2-9th plots are heatmaps for consensus clustering at k = 2–9. The rows and columns in the clustering heatmap are samples. **(B)** Cumulative distribution function (CDF). **(C)** Delta represents the relative change course in the area under the CDF curve when k = 2–9. **(D)** PCA results of the expression profiles of the two FRHGcluster patterns, showing the marked differences in the transcriptomes between the different FRHGclusters. The blue dots in the scatter plot represent FRHGcluster A, and the red dots represent FRHGcluster B.

We performed a consensus clustering analysis based on five Parkinson’s disease FRHGs expression matrices. The consensus matrix plot shows that the Parkinson’s disease sample can be clearly distinguished into two subtypes at K = 2 (Fig 7A). Consensus cumulative distribution function (consensus CDF) plots for K(2–9) show that the CDF distribution is flatter and near maximum for K = 2 (Fig 7B). The Delta area plot shows a large relative change in the area under the CDF curve for K = 2–4 (Fig 7C).

These results indicate that the fractal is most stable at k = 2. That is, Parkinson’s disease patients can be divided into two highly stable ferroptosis subtypes, which are named "FRHGcluster A" and "FRHGcluster B", respectively. Of these, 36 Parkinson’s disease samples were classified as FRHGcluster type A and 13 Parkinson’s disease samples were classified as FRHGcluster type B.

**Fig 8:**
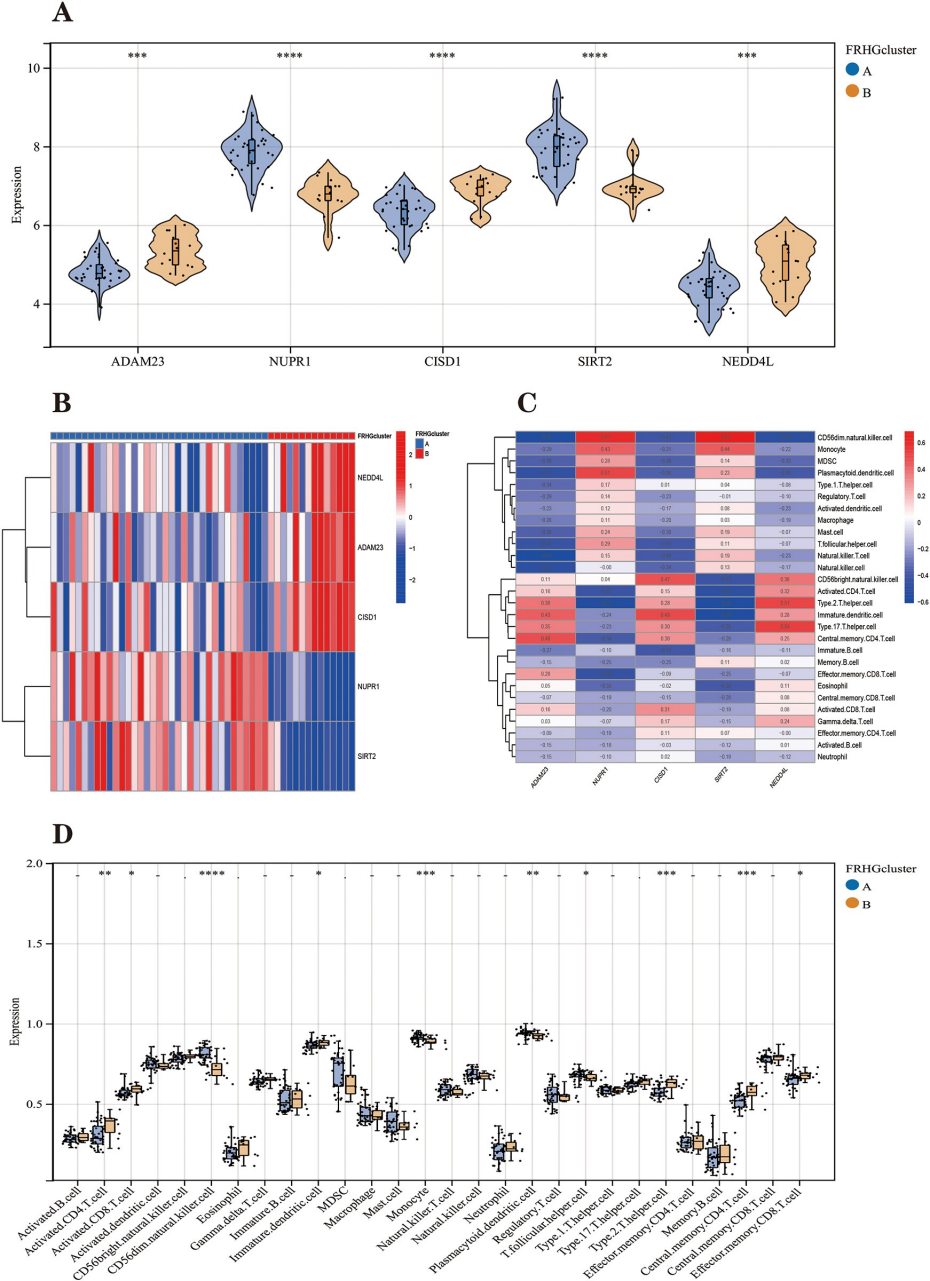
**(A)** Violin plot showing 5 FRHGs significantly differentially expressed in the two isoforms. **(B)** The heat map showed that the five FRHGs were differentially expressed in the two isoforms. **(C)** Heatmap of the correlation between Hub genes and 28 immune cells with a colour gradient change from red (positive correlation) to blue (negative correlation). **(D)** Comparison of the percentage of immune cell infiltration between FRHGcluster A and B. Blue represents FRHGcluster A; yellow represents FRHGcluster B. *P < 0.05; **P < 0.01; ****P < 0.001.

The PCA plot shows that the two Parkinson’s disease ferroptosis subtypes can be distinguished (Fig 7D). We clustered and plotted box lines and heat plots (Fig 8A and 8B) for 49 Parkinson’s disease samples, showing that Parkinson’s disease FRHGs showed significant differences in the two subtypes.

We quantified the degree of immune infiltration of 28 immune cells in Parkinson’s disease samples using the ssGSEA method (S5 Data) and used immune infiltration heat maps to visualize differences in the immune microenvironment between the two Parkinson’s disease ferroptosis subtypes. Fig 8D shows the immune characteristics of two ferroptosis subtypes. Plasmacytoid. dendritic.cells, T.follicular.helper.cells, CD56dim.natural.killer.cells, and Monocyte were higher in Parkinson’s disease FRHGcluster A than in FRHGcluster B. While Parkinson’s disease FRHGcluster A had higher proportions of Activated.CD4.T.cells, Activated.CD8.T.cells, Immature. dendritic.cells, Effector. memory.CD8.T.cells, Central. memory.CD4.T. cell and Type.2.T.helper.cell were lower than those of cluster B.

**Fig 9:**
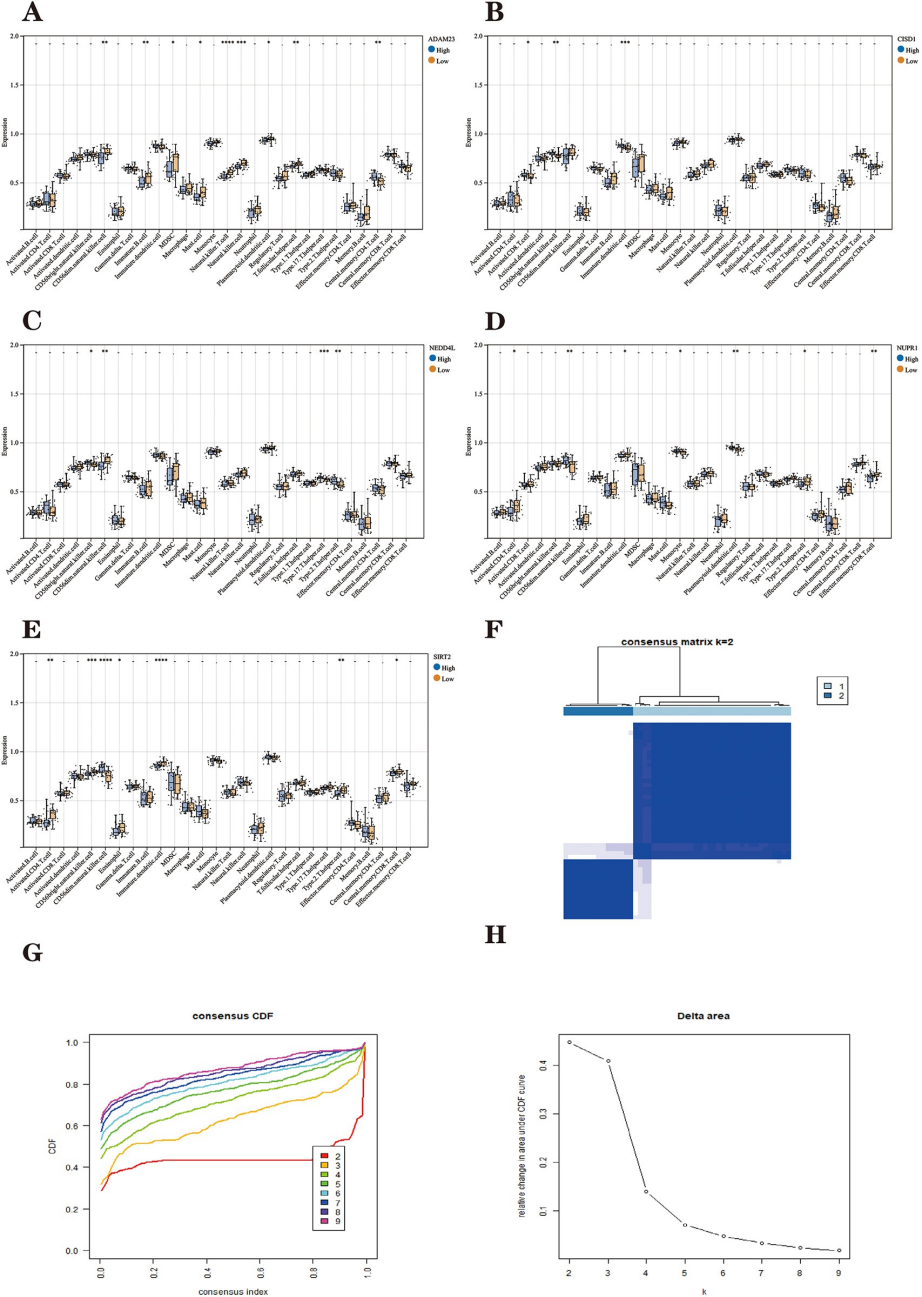
**(A)** Immune infiltration of ADAM23 at high and low expression. **(B)** Immune infiltration of CISD1 at high and low expression. **(C)** Immune infiltration of NEDD4L at high and low expression. **(D)** Immune infiltration of NUPR1 at high and low expression. **(E)** Comparison of the differences in immune cell abundance between the high and low expression groups for SIRT2. **(F)** The first plot indicates the color gradient from 0 to 1 (white: 0, blue: 1). The 2-9th plots are heatmaps for consensus clustering at k = 2–9. The rows and columns in the clustering heatmap are samples. **(G)** Cumulative distribution function (CDF). **(H)** Delta represents the relative change process of the area under the CDF curve when k = 2–9. *P < 0.05; **P < 0.01; ****P < 0.001.

The correlation between the five FRHGs and immune cells was further analyzed and visualized in the form of a heat map (Fig 8C). In addition, the differences in the degree of immune cell infiltration at the high and low expression of five Parkinson’s disease FRHGs were also analyzed. Fig 9A–9E shows that the level of gene expression is inextricably linked to the infiltration of immune cells.

#### Parkinson’s disease ferroptosis subtype differentially expressed genotypes have different immune environment characteristics

Differential expression analysis of genes related to the two ferroptosis subtypes revealed that 121 genes were significantly differentially expressed in the two subtypes (S6 Data). The threshold for differentially expressed genes was set to adj.p.value <0.05 and |log FC| >1. A new molecular typing based on differentially expressed genes in the ferroptosis subtype of Parkinson’s disease was established by unsupervised cluster analysis using the "ConensusClusterPlus" toolkit of R software.

**Fig 10:**
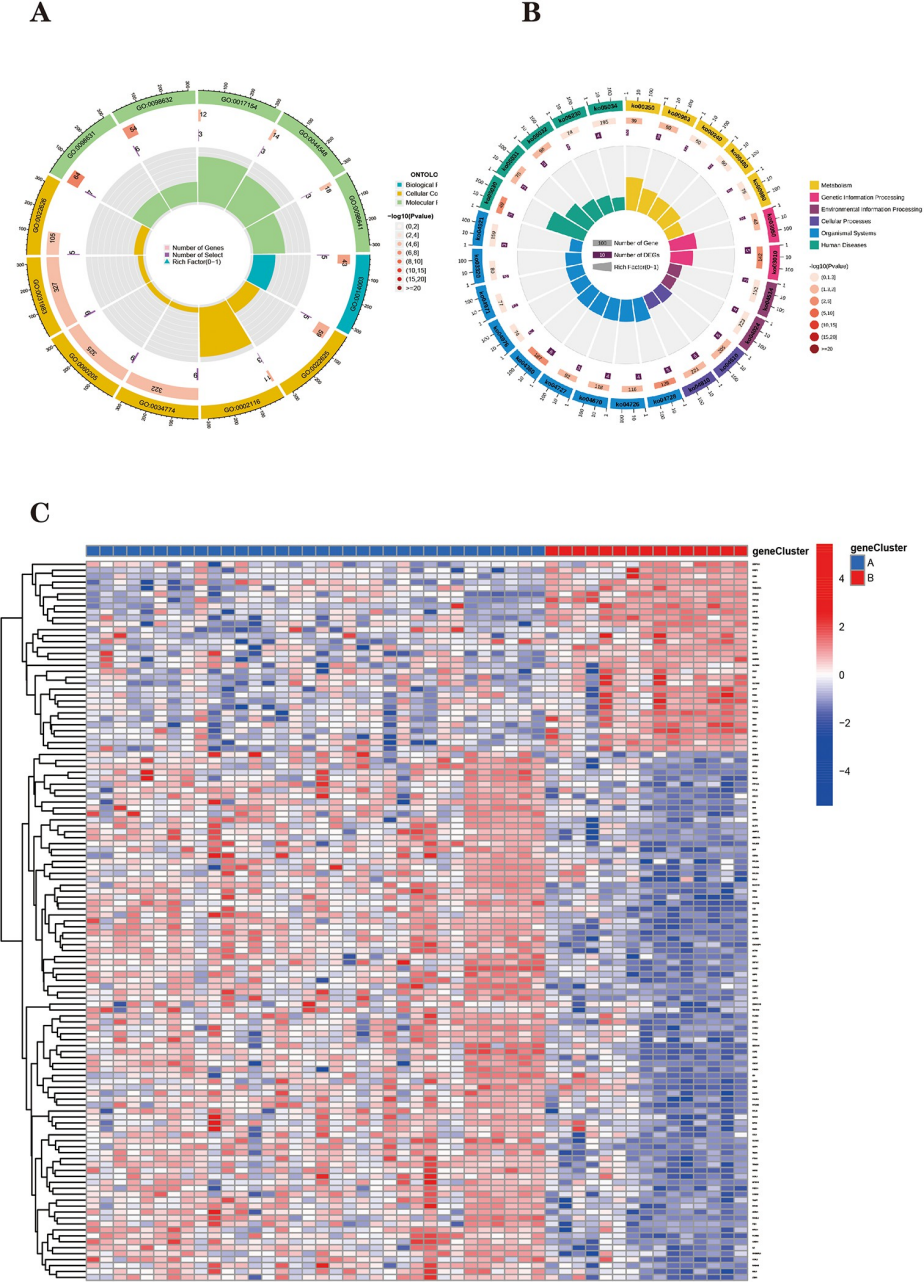
**(A, B)** The GO and KEGG functional enrichment analysis and the enrichment circle plot visualization results were used to understand the possible mechanism of the ferroptosis subtype differentially expressed genes in Parkinson’s Disease. **(C)** Heatmap showing the differential expression of ferroptosis subtype differentially expressed genes in geneCluster A and B.

**Fig 11:**
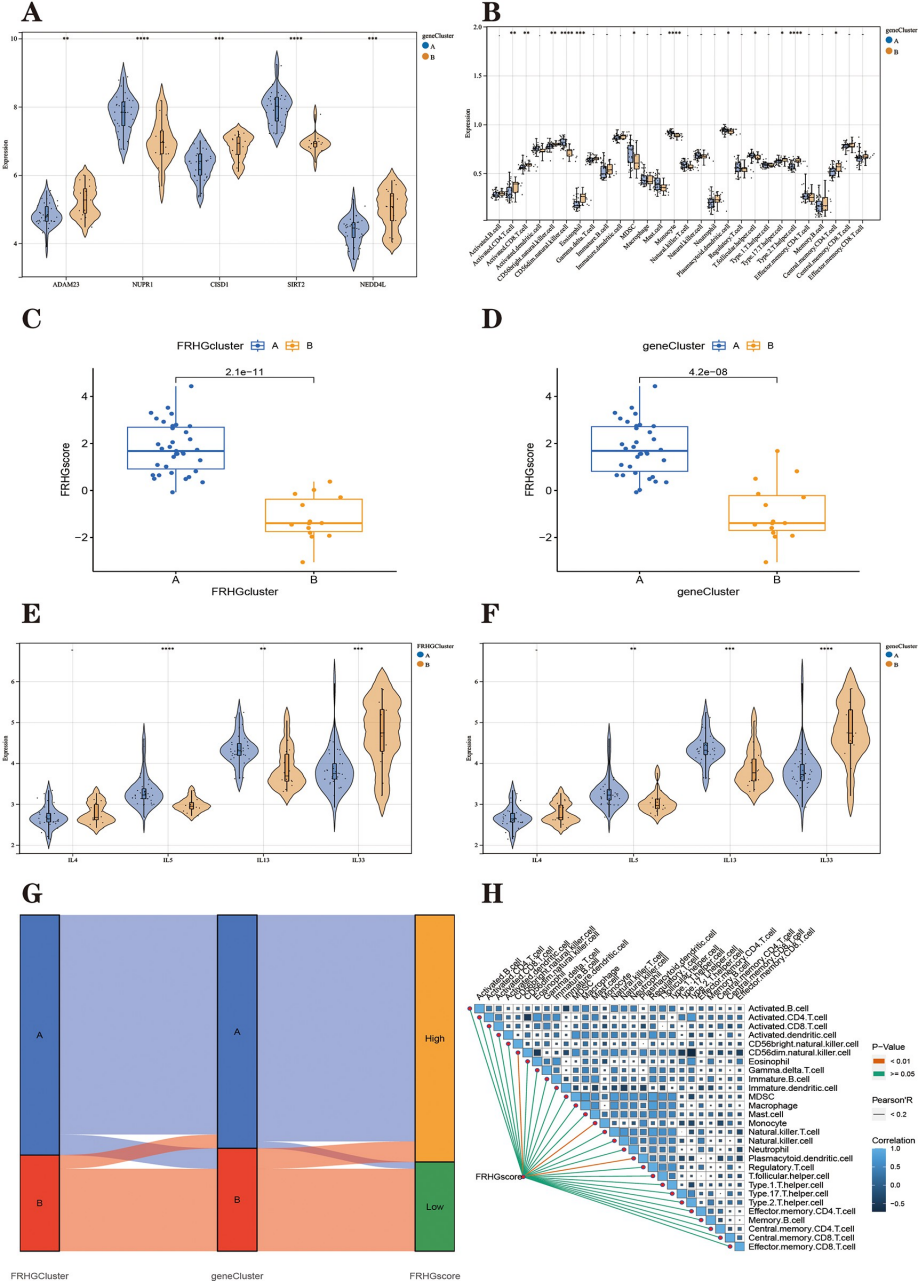
**(A)** Differential expression of FRHGs between genecluster A and B. **(B)** Comparison of the percentage of immune cell infiltration between geneCluster A and B; Blue represents FRHGcluster A, yellow represents FRHGcluster B. **(C)** The corresponding FRHGs score was obtained based on the PCA algorithm to compare the similarities and differences between the FRHGclusterA and B subtypes FRHGs score values. **(D)** The corresponding FRHGs score was obtained using the PCA algorithm to compare the similarities and differences between the geneCluster A and B subtypes FRHGs score values. **(E)** Comparison of differential expression of interleukins between FRHGcluster A and B; Blue represents FRHGcluster A, yellow represents FRHGcluster B. **(F)** Comparison of differential expression of interleukin between genecluster A and B. Blue represents geneCluster A, yellow represents geneCluster B. **(G)** Sanky plots indicate FRHGs score and molecular correlations. **(H)** In this figure, the yellow line represents P < 0.01, and the green line represents P ≥ 0.05; the colour of the squares represents the correlation coefficient, with navy blue being a negative correlation and light blue a positive correlation. *P < 0.05, **P < 0.01, ****P < 0.001.

The final PD patients were divided into two subtypes (Fig 9F–9H), named "geneCluster A" and "geneCluster B", respectively. Heat maps were plotted (Fig 10C) and it can be seen that the Parkinson’s disease ferroptosis subtype differentially expressed genes (32 upregulated in geneCluster A and 89 downregulated in genecluster B) are differentially expressed in geneCluster A and B. Fig 11A shows that FRHGs are differentially expressed in the two ferroptosis genomic subtypes. In addition, we used the ssGSEA algorithm to derive the extent of differences in the penetration of 28 immune cells in the ferroptosis genomic subtypes (Fig 11B).

#### Biological functional annotation of differentially expressed genes in ferroptosis subtypes of Parkinson’s disease

The possible functions and signaling pathways involved in the differentially expressed genes of ferroptosis subtypes in Parkinson’s disease were analyzed by GO and KEGG enrichment analysis [65]. The functions analyzed by GO are as follows: BP-related functions are mainly related to oligodendrocyte development; CC-related functions are mainly related to cytosolic large ribosomal subunit and semaphorin receptor complex. MF-related functions are mainly related to semaphorin receptor activity, cell adhesion mediator activity, S100 protein binding and intercellular adhesion. protein binding and cadherin binding involved in cell-cell adhesion (Fig 10A) (S7 Data). KEGG analysis showed that two Parkinson’s disease subtypes related genes are mainly involved in Drug metabolism—other enzymes, ribosomes, Focal adhesion and other pathways (Fig 10B) (S8 Data).

#### Correlation of FRHGs score with ferroptosis subtypes and ferroptosis genomic typing and immunological features

Ferroptosis-related hub genes score (FRHGs score) was calculated for each Parkinson’s disease sample based on PCA results (S9 Data). Based on the results of FRHGs scores obtained from the PCA, the differences in scores between subtypes were further analyzed. We found that FRHGclusterA had a higher FRHGs score than FRHGclusterB (Fig 11C); genecluster A had a higher FRHGs score than genecluster B (Fig 11D). The relationship between ferroptosis subtypes, ferroptosis genomic subtypes and FRHGs score were analyzed and their relationship was described using SAKY plots (Fig 11G). We also analyzed the relationship between ferroptosis subtypes, ferroptosis genomic subtypes and interleukin factors and found that IL5, IL13 and IL33 were differentially expressed in both ferroptosis subtypes and ferroptosis genomic subtypes, suggesting that interleukins may be a feature of both ferroptosis subtypes and that different interleukin factors are involved in PD progression in different subtypes (Fig 11E–11F). Further correlation analysis between FRHGs score and 28 immune cells showed that FRHGs score was significantly positively correlated with Monocyte, CD56dim.natural.killer.cell and Plasmacytoid. dendritic.cell (Fig 11H), while in the previous analysis showed that the expression of these immune cells was also higher in FRHGcluster A than in FRHGcluster B. FRHGcluster A also has a higher ferroptosis score than FRHGcluster B. This suggests that monocytes, CD56dim natural killer cells and plasmacytoid dendritic cells are more closely associated with ferroptosis in FRHGcluster type A and may be key target cells for immune association with ferroptosis. This also suggests that dysregulation of the immune microenvironment and ferroptosis play a key role in the development of PD.

### 2.3 Part three: Ferroptosis-related hub gene typing in Parkinson’s disease and its clinical relevance

#### Correlation analysis of FRHGs and immune characteristics

**Fig 12:**
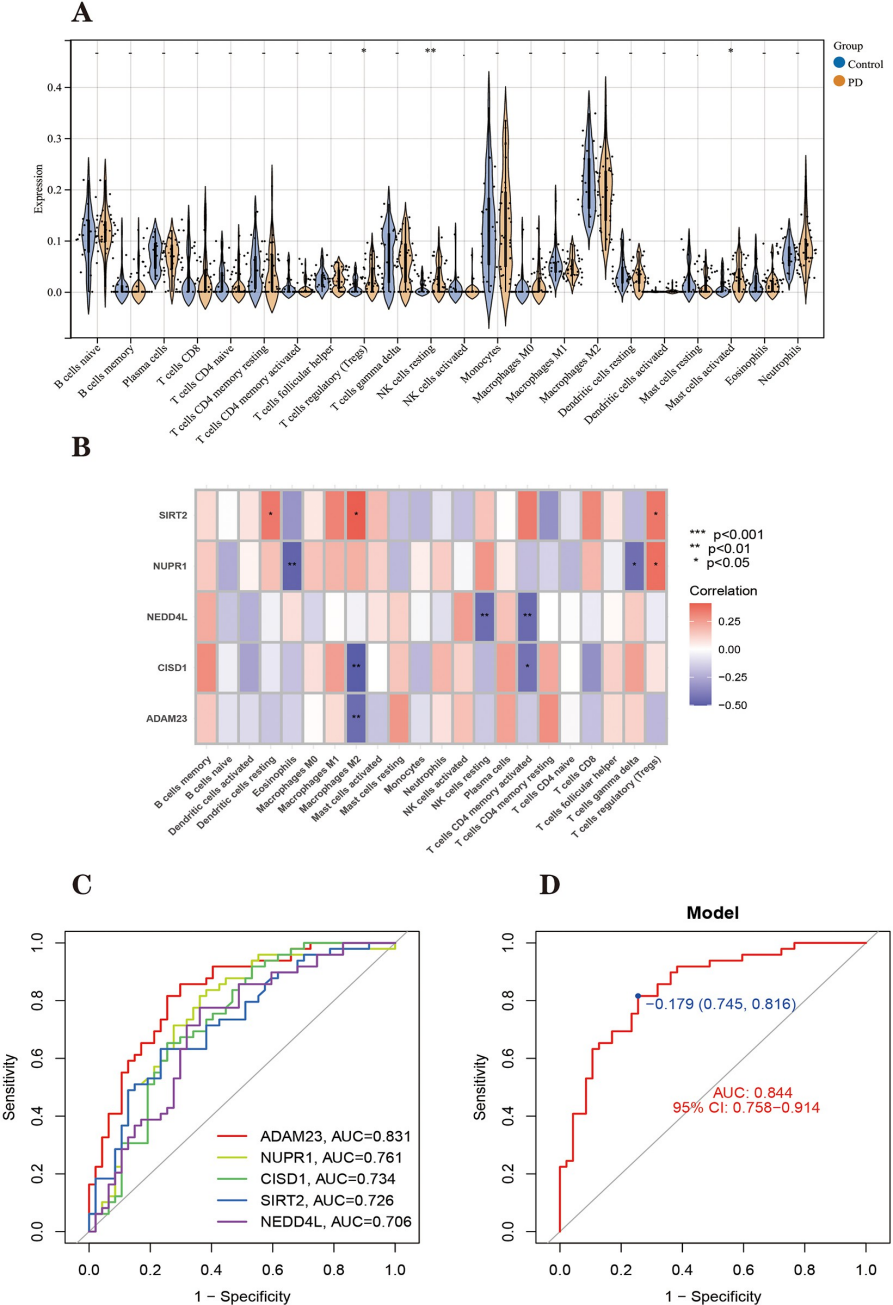
Immune infiltration landscape between PD and control obtained by CIBERSORT analysis. (A) Violin plot showing the difference in immune cell infiltration between PD (yellow) and Control (blue), *P* < 0.05, was considered statistically significant. (B) Shows the correlation between FRHGs and immune cells. The colors from red to purple represent the change from positive to negative correlations, respectively. More asterisks and darker colors of the modules represent stronger correlations. (C-D) ROC curves of the training dataset, (C) ROC curves of Hub genes, and (D) ROC curves of the model. *P < 0.05; **P < 0.01; ****P < 0.001.

We used an alternative immuno-permeation algorithm to explore the differences in immuno-permeation between PD patients and normal samples (S10 Data). As shown in Fig 12A, the proportions of Tregs (T cells regulatory), natural killer cells resting (NK cells resting) and mast cells activated (Mast cells activated) were higher in PD patients than in healthy patients (Control group). Further analysis of the correlation between FRHGs and 22 immune cell infiltrates (Fig 12B) showed that Dendritic cells resting were positively correlated with the SIRT2 gene; Eosinophils were significantly negatively correlated with NUPR1; Macrophages M1) were negatively associated with ADAM23 and CISD1 genes and positively associated with SIRT2 gene; NK cells resting was negatively associated with NEDD4L gene; T cells CD4 memory activated was negatively associated with CISD1 and NEDD4L gene; Tregs were positively correlated with SIRT2 and NUPR1 genes; and gamma delta T cells were negatively correlated with NUPR1. This suggests that alterations in the immune microenvironment of PD patients may be associated with these 5 FRHGs.

#### Parkinson’s disease FRHGs are closely associated with multiple PD-related pathways

**Fig 13:**
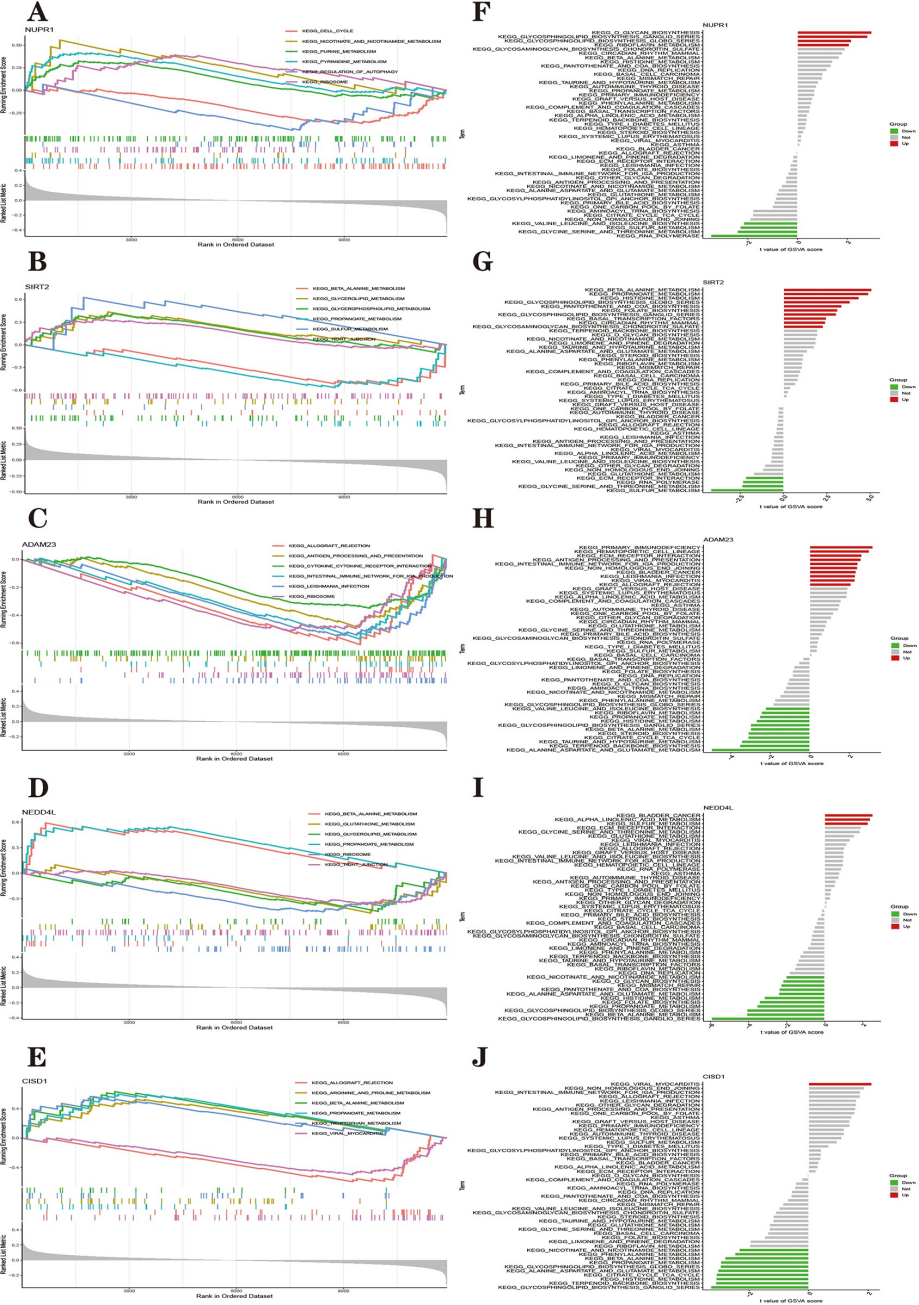
**(A–E)** Single-gene GSEA-KEGG pathway analysis in NUPR1 (A), SIRT2 (B), ADAM23 (C), NEDD4L (D), CISD1 (E). **(F–J)** High- and low-expression groups based on the expression levels of each marker gene combined with GSVA in NUPR1 (F), SIRT2 (G), ADAM23 (H). NEDD4L (I), CISD1 (J).

To further explore the role of FRHGs in PD in Parkinson’s disease, a single-gene gene set enrichment analysis (GSEA) pathway analysis was performed. Fig 13A–13E show the top six pathways enriched for each Parkinson’s disease FRHGs. We found that all five hub genes were enriched in the Propanoate metabolism pathway. CISD1, NEDD4L and SIRT2 are all enriched in the beta-Alanine metabolism pathway. NUPR1, NEDD4L and SIRT2 are all enriched in the ribosomal pathway. Both NEDD4L and SIRT2 are enriched in the Tight junction pathway, and both ADAM23 and CISD1 are enriched in the Allograft rejection pathway. Notably, FRHGs in Parkinson’s disease are enriched in multiple metabolic pathways, such as tryptophan metabolism, glutathione metabolism, and sulfur metabolism, suggesting that metabolism-related pathways play an important role in ferroptosis in Parkinson’s disease, which also involves complex metabolism of lipids, amino acids, and iron. In addition, some gene functions point to pathways such as Antigen Processing and Presentation and Leishmania Infection, further confirming the role of neuroimmune in PD. The specific enrichment results for each gene are integrated into S11 Data.

Further gene set variation analysis (GSVA) enrichment analysis of FRHGs in Parkinson’s disease was performed. The differences in activation pathways between the high and low gene expression groups were calculated and the results were visualized. Fig 13F–13J shows that low expression of CISD1, ADAM23 and NEDD4L genes and high expression of NURP1 and SIRT1 genes are associated with pathways such as Glycosphingolipid biosynthesis globo series. The low expression of CISD1 and NEDD4L genes and high expression of NURP1 and SIRT1 genes were associated with the expression of the Glycosphingolipid biosynthesis-ganglion series pathway. The low expression of CISD1, ADAM23 and NEDD4L and the high expression of SIRT1 was associated with the pathways of propanoate metabolism, beta-Alanine metabolism and histidine metabolism. Similarly, the results of GSVA analysis showed that high versus low expression of FRHGs was associated with multiple metabolic pathways, including sulfur metabolism, TCA cycle, and glycine metabolism.

#### Construction of a competitive endogenous lncRNA-miRNA-mRNA network

**Fig 14:**
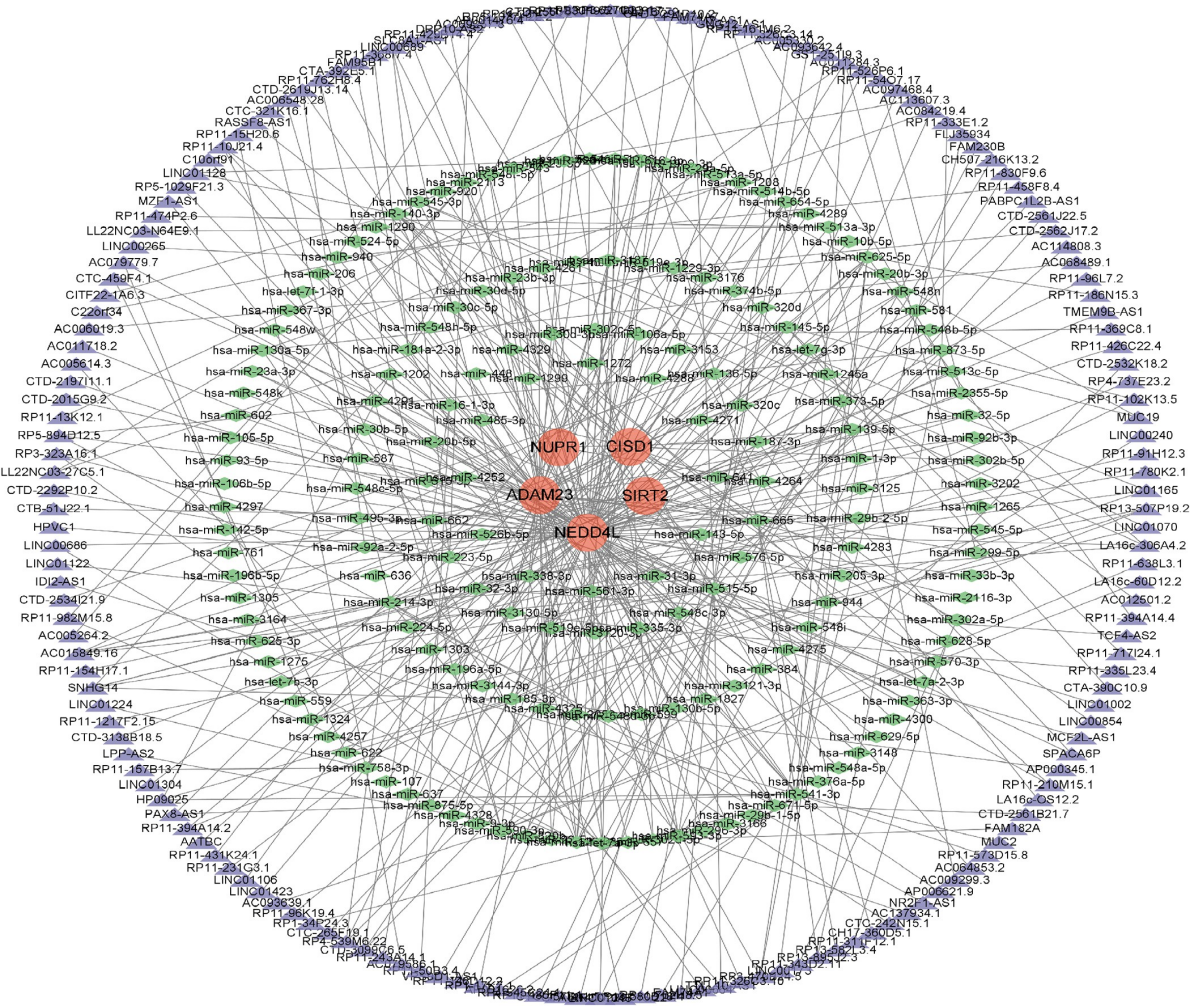
A ceRNA networks based on Hub genes. The network includes 351 nodes (5 mRNAs, 177miRNAs,169 lncRNAs), 421 edges. Red orbs represent Hub genes, green triangles represent miRNAs, and purple orbs represent lncRNAs.

The network includes 351 nodes (of which 5 hub genes, 177 miRNAs and 169 lncRNAs) and 421 edges (Fig 14). The specific details of the competing endogenous RNA (ceRNA) network are shown in S12 Data. According to Degree analysis, miRNAs such as has-miR-338-3p, hsa-miR-125a-3p, has-miR-515-5p, hsa-miR-665, has-miR-541-3p, and has-miR-214-3p may play important regulatory roles in the network. SNHG14, LA16c-306A4.2, AC011284.3, RP11-10J21.4 and AC015849.16 are lncRNAs that may play important regulatory roles in the network.

#### Validation of the accuracy of ferroptosis-related hub genes in Parkinson’s disease and classification of diagnostic models

We construct logistic regression classification diagnostic models based on FRHGs. The accuracy of the model and genes in the training set was evaluated using ROC curves as shown in Fig 12C and 12D. The area under the curve (AUC)of the model = 0.844. The AUC values of all five Parkinson’s disease FRHGs were greater than 0.7, indicating that all had some accuracy.

After that, three independent external datasets (GSE49036, GSE7621 and GSE26927) were used to validate the accuracy of FRHGs and their classification diagnostic models. In validating the accuracy of the model using an independent dataset, the AUC values of the five pivotal genes and the AUC values of the model were calculated in the same way.

**Fig 15:**
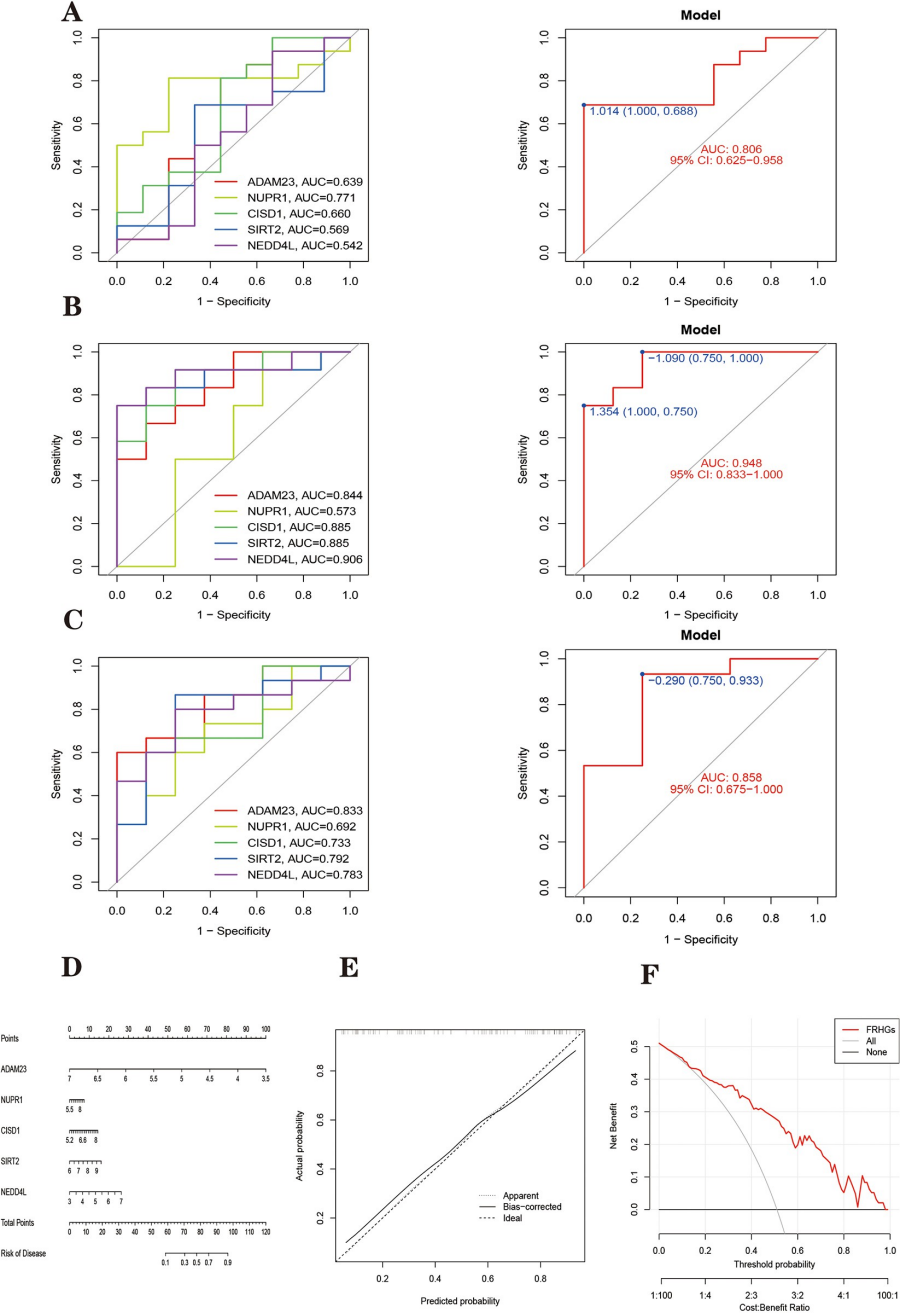
Logistic regression classification diagnosis model and risk prediction model. (A) The validation set GSE7621 dataset, (left) ROC curve of Hub gene, (right) ROC curve of the model. (B) The validation set GSE26927 (left) ROC curves of Hub gene, (right) ROC curves of the model. (C) The validation set GSE49036 (left) ROC curves of Hub gene, (right) ROC curves of the model. The different coloured lines represent different genes. (D) Establishment of a nomogram for predicting the risk of PD based on FRHGs. (E) The calibration curve evaluates the prediction efficacy of the nomogram. (F) DCA estimates the clinical benefit of the nomogram.

Where AUC = 0.806 for the GSE7621 validation model (Fig 15A). AUC = 0.948 for the GSE26927 validation model (Fig 15B). AUC = 0.858 for GSE49036 validation model (Fig 15C). The AUC values of the logistic regression classification diagnostic model were greater than 0.8 in both the training set and the three external independent datasets, indicating that the diagnostic model has good classification performance for PD samples and normal samples.

The AUC values of FRHGs in both the training set (Fig 12C) and the three validation sets (Fig 15A–15C) were greater than 0.5, indicating a certain degree of accuracy. And the fluctuation between different data is large, presumably due to the large effect of the error caused by the small number of samples in the validation set. We expect more large sample datasets to become available to corroborate the accuracy of these genes.

#### Development and validation of a risk prediction model for Parkinson’s disease

We constructed a Parkinson’s disease risk prediction model based on FRHGs and plotted a Nomogram for visualization (Fig 15D). Each trait gene included in the analysis corresponds to a scoring criterion. The total risk prediction score was obtained by summing the prediction scores of all trait genes. The proportional columns of risk prediction total scores correspond to corresponding Parkinson’s disease risks. The C-index of the model was 0.844, indicating that the prediction model has good risk prediction ability.

We also calculated the calibration values of the model, which showed Mean absolute error = 0.03, Mean squared error = 0.00103, and 0.9 Quantile of absolute error = 0.042. Plotting the calibration curve to visualize the above analysis, it can be seen that the model predicts a smaller deviation and higher agreement between the predicted and actual values (Fig 15E). This suggests that Nomogram mapping using five Parkinson’s disease ferroptosis-associated hub genes is more accurate in predicting Parkinson’s disease risk. In addition, analysis of the ROC curve and decision curve (Fig 15F) combining the training set showed a higher clinical benefit of the nomogram for patients with Parkinson’s disease.

## 3 Materials and methods

The study was carried out in three parts, and the technical route of the research method is shown in Fig 1A, and the specific details of the study are described in the subsequent method.

### 3.1 Part one: Screening for ferroptosis-related hub genes in Parkinson’s disease

#### Selection and acquisition of study datasets

The GEO database is the world’s largest database of non-oncology diseases and the largest publicly available gene chip database for Parkinson’s disease. We collated all human Parkinson’s disease nigrostriatal region gene microarray datasets in the GEO database and included those with a sample size greater than or equal to 20 in the study. Also, the human nigrostriatal Parkinson’s disease gene microarray dataset GSE42966, which contains the largest number of Braak stage 3–4 samples, was included in the study. The GSE42966 dataset contains a total of 15 samples, six from healthy human SN tissue and nine from Parkinson’s disease SN tissue (including four samples from Braak stage 3 and five samples from Braak stage 4). The details of each sample will be shown in Table 2.

**Table 2:** Dataset information from the GEO database.

Location	Accession	Platform	Type	Number
Brain	GSE8397	GPL96	Microarray	15 control vs. 24 PD
Brain	GSE20292	GPL96	Microarray	18 control vs. 11 PD
Brain	GSE20186	GPL96	Microarray	14 control vs. 14 PD
Brain	GSE49036	GPL570	Microarray	8 control vs. 15 PD
Brain	GSE7621	GPL570	Microarray	9 control vs. 16 PD
Brain	GSE26927	GPL6255	Microarray	8 control vs. 12 PD
Brain	GSE42966	GPL4133	Microarray	6 control vs. 9 PD

#### Data pre-processing and merging

First, the gene expression matrices of the seven datasets and their grouping information were obtained. Afterward, three datasets (GSE8397, GSE20292, and GSE20186) annotated using the GPL96 platform file were chosen to be combined and batch effects removed to maximize the elimination of batch effects due to technical biases arising from the processing and measurement of different datasets, while increasing the sample size of the study. Batch correction of the merged data was performed using the SVA toolkit of R software [66]. PCA plots were drawn to visualize the correction effect of the batch effect.

Ultimately, an expression matrix containing 47 healthy individuals and 49 nigrostriatal tissues from Parkinson’s disease patients was obtained. GSE42966 was used to analyze the association of FRGs in Parkinson’s disease with the progression of Braak stage 3–4 pathology. GSE49036, GSE7621 and GES26927 were used as independent external datasets to validate the accuracy of the obtained FRHGs and categorical diagnostic models for Parkinson’s disease.

#### Obtain the most relevant modules for PD and annotation of their biological functions

The combined Parkinson’s disease nigrostriatal tissue expression matrix was analyzed using the "WGCNA" toolkit in R software to explore gene expression and interactions in PD samples [48, 67]. First, the standard deviation of these genes was calculated and the top 25% of genes with the largest fluctuations were selected for analysis [48]. A scale-free co-expression network was constructed by calculating the Pearson correlation coefficient between every two genes [68]. The Pick-Soft-Threshold function is then used to calculate the adjacency values [69]. Second, the neighbor-joining values are converted into a topological overlap matrix (TOM) to measure the average network connectivity of each gene [70]. The dissimilarity between genes is calculated (1-TOM). Third, the topological overlap matrix is hierarchically clustered using an averaging algorithm to identify clusters of interrelated genes (i.e., modules). The minimum number of genes per module is limited to 80, and the deepSplit value is set to 2 [71]. Genes with similar expression profiles are divided into different modules using a dynamic tree-cutting method [72]. Fourth, the shear height is set to 0.05, and the modules below the shear height will be merged. We also calculated correlation coefficients and P-values between Module Eigengene (ME) values and clinical trait phenotypes for each module using Pearson correlation coefficients. Heat maps of correlations between modules and sample clinical traits were drawn. Then the correlation between the genes within the module and the module was calculated using the Pearson’s correlation coefficient, and the GS value for each gene and module was obtained; the correlation between the expression of the genes within the module and the first principal component of the module, i.e., ME, was calculated using Pearson’s correlation coefficient, and the MM value was obtained. Scatter plots of the GS and MM correlations of the modules were plotted [73]. The two modules with the highest positive and negative correlations with the Control group (healthy group) and the PD group were selected for further analysis. Genes with GS > 0.2 and MM > 0.8 were defined as module key genes [74]. To understand the possible biological functions of the obtained positive and negative correlation modules for Parkinson’s disease, we selected the most negatively correlated Yellow module and the most positively correlated Green module with PD for functional enrichment analysis. The screening condition was set as P value < 0.05 and Q value < 0.05. The KEGG analysis of these twomodules was performed using the OmicShare Tools (https://www.omicshare.com/tools) online analysis website, setting the screening condition to a P value < 0.05 [65, 75].

#### Identification of ferroptosis-related genes in Parkinson’s disease

The FerrDb database (http://www.zhounan.org/ferrdb/current/) collects the most recent ferroptosis-related genes, and all ferroptosis-related genes in this data have been experimentally validated [76]. After removing gene name duplications due to multiple functions at the same time, 484 FRGs were identified [77]. Genes that meet both the ferroptosis-related gene set and the key genes in the Parkinson’s disease key module were identified as FRGs in Parkinson’s disease [75, 77, 78]. A total of eight Parkinson’s disease FRGs were identified, including three genes negatively associated with PD and five genes positively associated with PD. The correlation between Braak staging of Parkinson’s disease and FRGs in the GSE42966 dataset was analyzed using the nonparametric Wilcoxon rank sum test [79].

#### Comparing two machine learning methods for screening ferroptosis-related hub genes in Parkinson’s disease

We constructed the training models using two machine learning methods, SVM (Support Vector Machine) and RF (Random Forests), respectively. Reverse cumulative distribution of residual, Boxplots of residual, and ROC was plotted to compare the accuracy of the two models [80]. The final choice was to construct a random forest model of FRGs in Parkinson’s disease using the RF algorithm to screen for FRHGs in Parkinson’s disease [81]. Random Forest is a machine learning algorithm proposed by Leo Breiman in 2001 [82]. Random forests can perform classification and regression tasks by building multiple decision trees. The essence of random forest is a classifier based on the ensemble learning method. The basic idea of ensemble learning is to combine multiple classifiers to achieve an ensemble classifier with better prediction effects. Random forests can also be used to evaluate the relative importance of features. Feature importance can help us filter features and thus hub genes to a certain extent [82].

The specific method is as follows. Set the tree value to 500 and calculate the number of decision trees required to achieve minimum error in cross-validation based on the expression matrix of Parkinson’s disease FRGs. After constructing the random forest model the Gini coefficient method was used to obtain the significant value scores of each dimension. The genes with the top 5 importance values were identified as FRHGs in Parkinson’s disease for subsequent analysis [83]. Intergenic correlation analysis of Parkinson’s disease FRHGs using Pearson’s correlation coefficient. Chromosome distribution localization analysis of Parkinson’s disease FRHGs was performed using the "RCircos" toolkit of R software.

### 3.2 Part two: Ferroptosis-related hub gene subtype in Parkinson’s disease and its clinical relevance

#### Parkinson’s disease FRHGs mediated by two subtypes of ferroptosis with different immune infiltration characteristics

We performed unsupervised consensus clustering analysis based on the expression profiles of five FRHGs using the "ConsensusClusterPlus" toolkit of R software on the combined 49 Parkinson’s disease samples to identify different Parkinson’s disease ferroptosis subtypes. Using the pam and euclidean algorithms as metric distances, 80% of the samples and 100% of the genes were selected for replicate sampling each time, and the number of replicate samples was set to 1000. The number of clusters was set from 2 to 9. Consensus Matrix, CDF and Delta area plots were drawn to show the effect of clustering with different K values to determine the optimal number of classifications. The new ferroptosis subtypes were named " Ferroptosis-related hub gene cluster A (FRHGcluster A)" and " Ferroptosis-related hub gene cluster B (FRHGcluster B)". The expression pattern and correlation of Parkinson’s disease FRHGs in ferroptosis subtypes were analyzed. PCA analysis of FRHGs in Parkinson’s disease was performed to demonstrate typing effects. To determine the role of the two Parkinson’s disease ferroptosis subtypes in the immune microenvironment, we applied the ssGSEA (single sample Gene Set Enrichment Analysis) algorithm to calculate the infiltration fraction of 28 immune cells contained in each Parkinson’s disease sample in the ferroptosis subtype [84]. The role of FRHGs in the immune microenvironment of Parkinson’s disease was analyzed and heat and box line plots were drawn [85]. The "GSVA" toolkit of the R software is used as a tool for ssGSEA analysis.

#### Identification of differentially expressed genotypes of ferroptosis subtypes with different immune environment characteristics

Differential expression analysis was performed between ferroptosis subtypes in Parkinson’s disease and genes with P < 0.05 and | log2FC| > 1 were identified as differentially expressed genes for ferroptosis subtypes [86]. The "ConsensusClusterPlus" toolkit of R software was applied to the combined 49 Parkinson’s disease samples to identify different differentially expressed genotypes of ferroptosis subtypes in Parkinson’s disease by unsupervised consensus clustering analysis. The new subtypes were named "geneCluster A" and "geneCluster B", respectively. The possible biological functions or pathways involved in the differentially expressed genes of ferroptosis isoforms were analyzed.

#### Analysis of the acquisition of ferroptosis score in Parkinson’s disease and its correlation with immune characteristics

To quantify the level of ferroptosis modifications in each individual, we established an evaluation index called ferroptosis-related hub genes score (FRHGs score) based on the expression profiles of five FRHGs in Parkinson’s disease [75]. This was done by first performing PCA using the prcomp function of the R software to assess the ability to differentiate between subtypes. PC1 and PC2 were then extracted to form signature scores. Ultimately, a method similar to the Genomic Grade Index (GGI) was applied to construct ferroptosis-related hub gene scores [87]. The specific formula is as follows:

FRHGscore=∑(PC1i+PC2i)

Where I denote the expression of ferroptosis-related hub genes.

The relationship between FRHGs scores and immune characteristics were also assessed using Pearson correlation analysis [68]. We also compared the typing with FRHGs score and multiple interleukin-related features, plotting Sanky plots for visualization. Pearson correlation analysis was also used to determine the correlation between FRHGs score and immune cells, and a correlation heat map was drawn to visualize the results.

### 3.3 Part three: Accuracy validation and correlation analysis of ferroptosis-related hub genes

#### Correlation analysis of FRHGs and the immune microenvironment in Parkinson’s disease

The CIBERSORT algorithm was used to calculate the penetration fraction of 22 immune cells for each sample in the combined dataset to obtain a matrix file of the proportion of 22 immune cells in each sample [88]. The correlation between FRHGs and 22 immune cells was then analyzed.

#### Development and validation of a diagnostic model for the classification of ferroptosis-related hub genes in Parkinson’s disease

A diagnostic model for the classification of Parkinson’s disease was developed based on previously obtained Parkinson’s disease FRHGs using a logistic regression algorithm. The accuracy of the Parkinson’s disease FRHGs and its classification diagnostic model were assessed using the AUC of the ROC [89, 90]. In addition, a total of three independent external datasets, GSE7621, GSE26927 and GSE49036, were used to validate the accuracy of hub genes and their classification diagnostic models.

#### Development and validation of a risk prediction model for Parkinson’s disease

A Parkinson’s disease risk prediction model was constructed based on the expression matrix of five Parkinson’s disease FRHGs using a logistic regression algorithm. Nomogram plots were plotted to visualize the risk prediction model of the model. The calibration of the model is calculated and calibration curves are plotted to visualize the range of deviation between the predicted and actual values of the model [91]. The clinical usefulness of the Parkinson’s disease risk prediction model was calculated and the benefit was visualized by drawing DCA curves using the "rmda" toolkit of R software [92].

#### Biofunctional annotation of ferroptosis-associated hub genes in Parkinson’s disease

GSEA (Gene Set Enrichment Analysis) can be used to elucidate whether genes are significantly different in the two biological states [79]. To further explore the potential mechanisms by which these five Parkinson’s disease ferroptosis hub genes affect PD, we performed a GSEA analysis for this purpose [93, 94]. Based on the expression matrix of the combined 96 samples, their correlation coefficients with other genes were calculated separately by spearman analysis based on the expression levels of ferroptosis hub genes in Parkinson’s disease. Meanwhile, "c2.cp.kegg.v7.5.1.symbols.gmt" was downloaded from the MSigDB database as a reference genome to check its abundance in the gene collection.

GSVA (Gene set variation analysis) is a non-parametric and unsupervised method for assessing the enrichment of transcriptomic gene sets [95]. The "GSVA" toolkit of R software was used to calculate and evaluate the obtained ferroptosis hub genes in Parkinson’s disease [96]. In addition, the "limma" package of the R program was used to calculate the difference in scores between overexpressed and below-normal expression samples to obtain the ferroptosis hub genes in Parkinson’s disease. The version of the reference gene set is "c2.cp.kegg.v7.5.1.symbols.gmt". Statistically significant pathways were determined at a P value < 0.05. Boxplots comparing differential expression levels of immune cells or genes between the two groups were calculated using the nonparametric rank-sum test (Wilcox.test).

#### Construction of a competitive endogenous RNA regulatory network for ferroptosis hub genes in Parkinson’s disease

Prediction of mRNA-miRNA interactions based on three public databases (miRanda, TargetScan, miRDB) using perl software [97]. The predicted miRNAs were then searched using perl software based on the SpongeScan database to obtain the corresponding miRNA-lncRNAs. The obtained lncRNA-miRNA-mRNA interaction network was imported into Cytoscape to map the ceRNA network.

## 4 Discussion

Ferroptosis, an iron-dependent programmed cell death associated with LPO, plays a crucial role in the pathogenesis of PD. On the one hand, several studies have confirmed that multiple pathological changes in the SN of PD patients are associated with ferroptosis. For example, α-syn functions in the ferroptosis pathway and iron chelators, D-PUFAs and iron inhibitors all inhibit the pathological aggregation of α-syn. The PARK7 gene encodes a DJ-1 protein that regulates ferroptosis. On the other hand, several studies have shown that immunity is involved in the development of PD. The immune pathway has also been shown to induce cell death through ferroptosis. In addition, it has also been found that the associated molecular patterns generated during ferroptosis (e.g. ROS) can induce microglia activation through the activation of neuroimmune pathways, ultimately leading to the development of neuroinflammation. This suggests that ferroptosis and neuroimmune responses are important components of the pathogenesis of Parkinson’s disease. However, the exact molecular mechanisms are not known. We need to deeply analyze the molecular patterns related to ferroptosis and its immunological characteristics, which support the study of pathogenic mechanisms of PD and the prediction of relevant drug targets, and provide a theoretical basis for complementary PD biomarkers.

We next obtained the two most relevant modules to PD by WGCNA, and the related functional analysis showed that the function of the positive correlation module (Green module) was enriched to the ferroptosis pathway and ferroptosis-related metabolic pathways such as cysteine and glutathione, in addition to the pathogenesis-related pathway of PD. The enrichment analysis of the negative module (Yellow module) showed that the module was mainly enriched in PD-related pathogenic pathways, including the synaptic vesicle cycle and dopaminergic synapses, but also in ferroptosis-related pathways such as metal ion channels and endocytosis.

We obtained eight Parkinson’s disease ferroptosis-related genes (MAP3K11, SNX4, SIRT2, NUPR1, ACSL4, CISD1, ADAM23 and NEDD4L) by taking intersections of key genes of these two PD modules with ferroptosis-related genes.

For the first time, ACSL4 and SNX4 expression was found to be increased in the Braak4 phase compared to the Braak3 phase. There are two common staging of Parkinson’s, one is the Hoehn-Yahr staging (H-Y staging) which is most commonly used clinically, and one is the pathological staging proposed by Professor Heiko Braak in Germany: Braak staging, which is divided into 6 stages. According to Braak’s staging, the typical clinical symptoms appear only when the loss of nigrostriatal dopaminergic neurons is severe (stage 4) [98].

Therefore, GSE42966 data possessing Braak stages 3–4 were selected for the study, and the results of the analysis suggest that these two genes may be involved in the progression of Braak3 to Braak4 case staging in Parkinson’s disease. ACSL4 plays a role in promoting (Polyunsaturated Fatty Acids)PUFAs incorporation into membrane lipids and ferroptosis pathways, and has been shown in several studies to reduce OS and ferroptosis in dopaminergic neurons in PD by targeting this gene for inhibition [99, 100]. In previous studies, ACSL4 has been shown to be involved in a variety of diseases such as rectal cancer, bladder cancer, chronic obstructive pulmonary disease, acute kidney injury, and non-alcoholic steatohepatitis through the ferroptosis pathway [61, 101–104]. Because ACSL4 can esterify PUFAs as a substrate for lipid peroxidation, which further triggers ferroptosis, and because polyunsaturated fatty acids play a crucial role in neuronal function, ACSL4 is considered to be a key gene in the pathogenesis of a variety of neurological disorders, including ischemic stroke and multiple sclerosis [105]. Recent studies have also confirmed that ferroptosis play a role in Alzheimer’s disease [106]. Qi Y et al. found that thiazolidinedione as an ACSL4 inhibitor ameliorated neuroinflammation and ferroptosis in a preclinical model of AD and reduced AD risk [107]. The present study also confirms at the genetic level that ACSL4 may promote the progression of key pathological stages of Parkinson’s disease through ferroptosis.

Recent studies have shown that sequenced connexin 4 (SNX4) is a synaptic protein whose altered protein levels are associated with Alzheimer’s disease. Overexpression of SNX4 significantly increased the levels of BACE1 and Aβ [108]. The down-regulation of SNX4 has the opposite effect. SNX4 interacts with BACE1 and prevents the transport of BACE1 to the lysosomal degradation system, resulting in a prolonged half-life of BACE1 and increased Aβ production [109]. Autophagy defects are associated with many human diseases, especially neurodegenerative diseases, inflammatory diseases, and cancer [110].

SN4 belongs to a family of proteins associated with endosomal sorting in the nucleus, and another protein of this family, SNX5, has been shown to promote ferroptosis in PD in both mouse and cellular models [111–113]. SNX4 and SNX5, on the other hand, can form a heterodimer that recognizes autophagosomal membrane proteins and is required to generate membrane curvature on autolysosomes [114]. The main pathological feature of PD is the pathological aggregation of α-syn, which can disrupt autophagy by inhibiting the required association of SNX4 with phagocytes [115]. And our study showed that SNX4 levels were increased in the Braak4 stage compared to the Braak 3 stage. Taken together, these studies led us to hypothesize that in PD, increased α-syn disrupts SNX4-mediated autophagy, increased SNX4 levels promote ferroptosis in dopaminergic neurons, and decreased cellular autophagy exacerbates the pathologic aggregation of α-syn.

We first tested the accuracy of two machine learning-built models and finally chose to use the random forest to obtain five Parkinson’s disease FRHGs (CISD1, SIRT2, NUPR1, ADAM23 and NEDD4L). Among them, CISD1, SIRT2 and NEDD4L have been experimentally confirmed to play a role in PD, but NUPR1 and ADAM23 have never been studied to confirm their association with PD [116–118].

SIRT2 (Sirtuin 2) has been shown to mediate dopaminergic neuronal loss in PD, and SIRT2 knockdown effectively ameliorates abnormal behavioral phenotypes in a transgenic mouse model of PD [119]. It has been found that SIRT2 regulates neuronal death during PD progression through Cyclin Dependent Kinase 5 (CDK5)-dependent nucleoplasmic shuttling. However, it has also been suggested that SIRT2 achieves neuroprotective effects by inhibiting ferroptosis. However, the mechanism of SIRT2-mediated ferroptosis in PD is unclear [120].

CISD1, also known as MitoNEET, is an iron-containing mitochondrial outer membrane protein involved in iron export from mitochondria. Knockdown of CISD1 exacerbated Erastin toxicity, increased mitochondrial iron content and LPO, and promoted ferroptosis, whereas stabilization of CISD1 attenuated Erastin toxicity and decreased mitochondrial LPO. Geldenhuys et al. have shown that loss of CISD1 leads to mitochondrial dysfunction and loss of striatal dopamine and tyrosine hydroxylase, promoting the progression of PD and exacerbation of symptoms [117]. These studies all confirm the involvement of CISD1 in PD progression through ferroptosis.

NEED4L (Neuronally Expressed Developmentally Downregulated 4 L) was found to ubiquitinate α-syn and promote its degradation [121]. NEDD4L-mediated COX4 degradation promotes OS and the development of neurodegenerative diseases [122]. However, in the study of the ferroptosis mechanism, NEDD4L was found to promote ferroptosis through the ubiquitination of SLC7A11 and GPX4 [123, 124]. There is a lack of studies on the mechanism of ferroptosis by NEED4L in the SN of PD. The present study showed that NEED4L was negatively correlated with PD.

NUPR1 (Nuclear protein 1) has been shown to be a key inhibitor of ferroptosis [125]. The specific mechanism is that NUPR1 blocks ferroptosis cell death by mediating LCN2 expression to reduce iron accumulation and subsequent oxidative damage. Other studies have also identified NUPR1 as a key regulator of the antioxidant system [125]. The present study also suggests that NUPR1 is involved in PD, is positively correlated with PD, and is most likely involved in PD pathogenic mechanisms through ferroptosis and OS.

Overexpression of ADAM23 (ADAM Metallopeptidase Domain 23) has been experimentally shown to promote ferroptosis in esophageal squamous cell carcinoma and is expected to play a role in future cancer therapy [126]. In contrast, the present study found that ADAM23 was negatively correlated with PD, and the specific mechanism of action needs to be investigated.

We further analyzed the functions of Hub genes by combining GSEA and GSVA algorithms. GSEA and GSVA analysis revealed that these genes are basically associated with various metabolic pathways (e.g. glutathione, triglyceride and sulfur metabolism), etc., while lipid, amino acid and sulfur metabolism are closely related to ferroptosis; in addition, they are also enriched to ribosomes, and plasmapheresis showed that the ribosomal pathway is the most relevant pathway to PD [127]. Almost all hub genes are enriched to the sphingolipid pathway. Sphingolipids are a well-defined subclass of lipids that regulate key aspects of brain function. One study found that sphingolipids are potent regulators of inflammatory processes. The dysregulation of sphingolipid metabolism in Parkinson’s disease is supported by a large body of evidence [128]. A key molecular mechanism of sphingolipid control of neuroinflammation in Parkinson’s disease [129]. These include inflammasome activation and secretion of pro-inflammatory cytokines, alterations in calcium homeostasis, changes in blood-brain barrier permeability, recruitment of peripheral immune cells or autoantibody production [130]. All five FRHGs are associated with neuroimmune-related pathways, and the sphingolipid pathway may be a bridge between PD ferroptosis and neuroimmune, which requires subsequent validation.

In further analysis of FRHGs in Parkinson’s disease, samples from patients in the PD group were divided into FRHGcluster A and FRHGcluster B by unsupervised clustering analysis. Quantification of the degree of immune infiltration of 28 immune cells in Parkinson’s disease samples using the ssGSEA method. We found a higher proportion of Plasmacytoid. dendritic.cells, T.follicular.helper.cells, CD56dim.natural.killer.cells and Monocyte in FRHGcluster A than in FRHGcluster B. While Parkinson’s disease FRHGcluster A had higher proportions of Activated.CD4.T.cells, Activated.CD8.T.cells, Immature. dendritic.cells, Effector. memory.CD8.T.cells, Central. memory.CD4.T. cell and Type.2.T.helper.cells were lower than those of cluster B. In addition, the differences in scores between subtypes based on PCA-derived FRHGs score results were further analyzed.

Neuroinflammation is involved in PD development through numerous inflammatory factors such as interleukins [131]. Single nucleotide polymorphisms of interleukin 13 (IL-13) and its receptor α1 (IL-13Rα1) in sporadic PD have been found to increase cellular susceptibility to OS and to increase the cytotoxic activity of IL-13 on human SH-SY5Y neurons exposed to sublethal doses of hydrogen peroxide, tert-butyl hydroperoxide, or the seed ferroptosis inducer RLS3 [132]. In addition, IL-13 has been found to stimulate the production of brain-derived neurotrophic factors by primary astrocytes, thereby promoting cognitive function [133]. Serum levels of IL-5 have been found to be generally reduced in PD patients. Several studies have found elevated concentrations of IL-33 in serum and midbrain and striatum in PD groups, and IL-33 enhances glial maturation factor (GMF)-mediated neuroinflammation [134, 135]. In addition, Xu et al. found that serum IL33 concentrations were higher in early PD patients than in late [136].

## 5 Conclusion

We synthesized multiple algorithms to comprehensively analyze ferroptosis-related molecules and their immunological features in Parkinson’s disease at the genetic level. In our study, we identified for the first time that ACSL4 and SNX4 may be associated with the Braak3 to Braak4 pathological grade progression of PD and are expected to be intervention targets for alleviating PD progression to the clinical symptom stage. We combined WGCNA and random forest algorithm to obtain two new FRHGs (NUPR1 and ADAM23) in Parkinson’s disease, which are expected to be new PD biomarkers and drug action targets. Based on hub genes, we developed a PD risk prediction model and a PD classification and diagnosis model using a logistic regression algorithm. Two different ferroptosis subtypes (FRHGcluster A and FRHGclustre B) were identified to help us adopt a more flexible treatment plan in the clinic for PD patients with different subtypes. The hub gene has helped us to construct a ceRNA regulatory network that enables us to better understand the molecular regulatory mechanisms of PD and reveal possible targets for drug action and risk prediction. We also found that IL-5, IL13, and IL33 were differentially expressed in the two ferroptosis gene isoforms, which may serve as markers to discriminate between the two isoforms. We also calculated the ferroptosis score for each sample. The FRHGs score was also found to correlate with both ferroptosis typing and immune pathways. Our study provides new insights into the role of ferroptosis in PD and its molecular immune mechanisms, as well as support for the discovery of new biomarkers and targets of action in PD at the genetic level.

## Supporting information

S1 Data GO analysis results of the yellow module. (XLS)

S2 Data KEGG analysis results of the yellow module. (XLS)

S3 Data GO analysis results of the green module. (XLS)

S4 Data KEGG analysis results of the green module. (XLS)

S5 Data Degree of immune infiltration of 28 types of immune cells. (XLS)

S6 Data Differential expression analysis results between two ferroptosis subtypes. (XLS)

S7 Data GO analysis results of DEGs among PD ferroptosis subtypes. (XLS)

S8 Data KEGG analysis results of DEGs among PD ferroptosis subtypes. (XLS)

S9 Data PCA calculation FRHGs score. (XLS)

S10 Data CIBERSORT algorithm calculates immune infiltration of 22 types of immune cells. (XLS)

S11 Data GSEA enrichment analysis results. (XLSX)

S12 Data The specific details of the ceRNA network. (XLS)
